# Exploring the Potent Anticancer Activity of Essential Oils and Their Bioactive Compounds: Mechanisms and Prospects for Future Cancer Therapy

**DOI:** 10.3390/ph16081086

**Published:** 2023-07-31

**Authors:** Fatouma Mohamed Abdoul-Latif, Ayoub Ainane, Ibrahim Houmed Aboubaker, Jalludin Mohamed, Tarik Ainane

**Affiliations:** 1 Medicinal Research Institute, Center for Studies and Research of Djibouti, IRM-CERD, Route de l’Aéroport, Haramous, Djibouti City P.O. Box 486, Djibouti; mohamed.jalludin@gmail.com; 2 Superior School of Technology of Khenifra, University of Sultan Moulay Slimane, P.O. Box 170, Khenifra 54000, Morocco; a.ainane@usms.ma (A.A.); t.ainane@usms.ma (T.A.); 3 Peltier Hospital of Djibouti, Djibouti City P.O. Box 2123, Djibouti; ibrahimhoumed@yahoo.fr

**Keywords:** essential oils, cancer, anticancer activity, cytotoxic activity, anticancer mechanisms

## Abstract

Cancer is one of the leading causes of death worldwide, affecting millions of people each year. Fortunately, the last decades have been marked by considerable advances in the field of cancer therapy. Researchers have discovered many natural substances, some of which are isolated from plants that have promising anti-tumor activity. Among these, essential oils (EOs) and their constituents have been widely studied and shown potent anticancer activities, both in vitro and in vivo. However, despite the promising results, the precise mechanisms of action of EOs and their bioactive compounds are still poorly understood. Further research is needed to better understand these mechanisms, as well as their effectiveness and safety in use. Furthermore, the use of EOs as anticancer drugs is complex, as it requires absolute pharmacodynamic specificity and selectivity, as well as an appropriate formulation for effective administration. In this study, we present a synthesis of recent work on the mechanisms of anticancer action of EOs and their bioactive compounds, examining the results of various in vitro and in vivo studies. We also review future research prospects in this exciting field, as well as potential implications for the development of new cancer drugs.

## 1. Introduction

Cancer is a complex disease that can take many forms [1]. Approximately 200 types of tumors can affect all tissues of the body [2], each resulting from the acquisition of abnormal characteristics by cells, such as independence from growth signals [3], resistance to growth-inhibiting signals, resistance to programmed cell death, the acquisition of unlimited replicative potential, the ability to induce the genesis of blood vessels or angiogenesis and the ability to form metastases [4]. These six properties, which vary between tumors, result from alterations in the expression or sequence of oncogenes and tumor suppressor genes [5].

According to a World Health Organization report published in 2008, the number of cancer cases worldwide had doubled over the past 30 years, reaching 12 million new cases and resulting in seven million deaths [6]. In 2020, cancer was responsible for nearly 10 million deaths, accounting for nearly one in six deaths and being one of the leading causes of death globally [7]. Projections for 2030 indicate a continued increase to 11.4 million. Although the growth of cancer therapies is considerable, the study of natural products is a promising research strategy for the discovery of new drugs [8]. Moreover, two-thirds of the drugs used in the treatment of cancer are of natural origin or derived from natural products [9].

Aromatic and medicinal plants play a key role in chemical, biological and pharmacological activities in general [10]. For thousands of years, they have been used to treat various diseases and ailments, thanks to their healing and therapeutic properties [11]. In addition, many medicinal plants are used as a source of inspiration in the synthesis of modern drugs [12]. Scientists study the properties of plant extracts to identify the active compounds that give them their therapeutic properties [13]. These compounds can then be synthesized in the laboratory to produce more effective and safer drugs. Essential oils (EO) are natural complexes of volatile and fragrant molecules synthesized by aromatic plants as secondary metabolites. They possess antimicrobial, antioxidant, anti-inflammatory, anti-proliferative and anti-cancer properties, particularly due to the presence of bioactive terpene and phenolic chemical compounds [14]. New research has brought attention to the potential of essential oils and their chemical components in combating cancer. However, further investigation is required to understand the specific physiological and molecular mechanisms responsible for their anti-tumor properties. [15].

### 1.1. Essential Oils

Essential oils (EOs) are complex natural organic compounds, exhibiting a variety of organic structures [16]. The term “oils” is used to describe their ability to dissolve in fats, while the term “essential” is used to denote the distinctive odor produced by the plants that produce them. [17]. EOs are secondary metabolites biosynthesized by aromatic plants, which contain specialized structures for their secretion, such as secretory hairs (in Lamiaceae), secretory pockets (in Myrtaceae) and secretory ducts (in Apiaceae). These structures vary depending on the plant organ and are also involved in EO storage [18].

EOs are mainly extracted by distillation, but there are other techniques such as cold extraction, supercritical carbon dioxide, and ultrasonic- or microwaveassisted extraction [19]. EOs are generally liquid at room temperature, volatile, flammable, and fragrant, and have a density generally less than one [20]. Some EOs have a characteristic color, such as blue tansy, red-brown cinnamon, or green Inula. They are insoluble in water but soluble in vegetable oils and most organic solvents, such as alcohol and ether. EOs can be oxidized rapidly and undergo isomerization under the effect of light [21].

The synthesis of essential oils (EOs) occurs through two primary pathways: the mevalonic acid (MVA) pathway, which occurs in the cytoplasm, mitochondria, and endoplasmic reticulum; and the deoxyxylulose phosphate or methylerythritol (MEP) pathway, which takes place in plastids. Depending on the pathway followed, the constituents of essential oils predominantly belong to two distinct biosynthetic families: terpenoids and phenylpropanoids. Monoterpenes (consisting of 10 carbons) and sesquiterpenes (consisting of 15 carbons) are the most commonly found compounds in EOs, although diterpene constituents may also be present [22,23].

The precise function of EOs in plants remains somewhat undefined; however, research has demonstrated several roles they play. EOs have been found to attract animals involved in pollination and seed dispersal, act as a defense mechanism against phytopathogenic organisms, and exhibit allelopathic effects [24].

### 1.2. Chemical Composition

Essential oils can contain approximately up to about 300 different molecules, but most of them contain between 20 and 60 molecules. Terpene compounds constitute the majority of these molecules, but there are also phenylpropanoids as well as other compounds such as nitric and sulfuric compounds, although their frequency is lower and proportion similar. Additionally, some essential oils may also contain nitrogen and sulfur compounds [25,26,27,28].

### 1.3. The Essential Groups of Essential Oils

**Figure 1:**
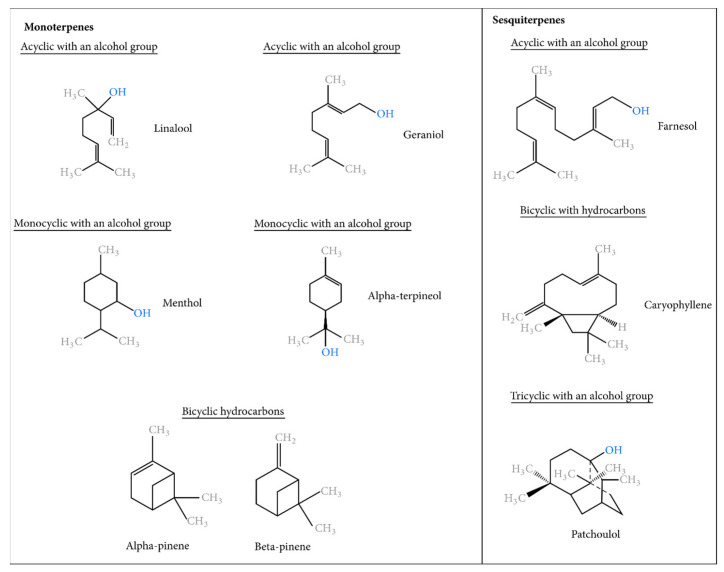
Some chemical structures of essential oil constituents.

Terpenes and terpenoids are the largest groups, with nearly 3000 terpenes described in the literature [29]. They include monoterpenes (10 carbon atoms in the molecule), sesquiterpenes (15 carbon atoms), and diterpenes (20 carbon atoms) [30]. Terpenes are organic molecules made up of a multiple of five carbon atoms, with a general formula of (C_5_H_8_)_n_. The base molecule is isoprene [31]. In its reactive form, isoprene is in the form of isoprenoylpyrophosphate (IPP), which partially converts to dimethylallylpyrophosphate (DMAPP). The compounds IPP and DMAPP react together to form geranylpyrophosphate (GPP), a precursor of C_10_ monoterpenes. A second molecule of PPI reacting with GPP provides farnesylpyrophosphate (FPP), a precursor of C_15_ sesquiterpenes [32]. A third PPI molecule reacting with FPP provides geranylgeranylpyrophosphate (GGPP), a precursor of C_20_ diterpenes. This process continues for the formation of C_25_ sesterpenes, C_30_ triterpenes, and C_40_ carotenes [33]. The boiling point of terpenes increases with the number of carbon atoms in the molecule, which means higher mass molecules are less volatile [34]. It is important to mention that essential oils are typically obtained through steam distillation or hydro-distillation methods, resulting in a high concentration of monoterpenes. However, they contain comparatively lower levels of diterpenes and even less triterpenes (Figure 1). [35,36].

The phenylpropanoid group is a less common set of phenylpropane derivatives (C_6_H_5_-CH_2_-CH_2_-CH_3_). The precursor of this series is shikimic acid (or trihydroxy-3,4,5-cyclohexane-1-carboxylic acid), which leads to the cinnamic acid derivatives C_6_H_5_-CH=CH-COOH. This second group includes aldehydes (like cinnamaldehyde) and methoxylated derivatives, as well as allylphenols (like eugenol) and propenylphenols (like anethole). Lactones or cyclic esters (like coumarin) can also form from cinnamic acid derivatives. Sometimes the aliphatic chain is reduced to a single carbon atom, as is the case with vanillin (Figure 1) [37,38].

Additionally, essential oils often contain nitric and sulfuric compounds as a result of the degradation of molecules with low or no volatility [39]. For instance, the oxidation of linoleic and linolenic acids generates unstable peroxides that, upon further degradation, give rise to alcohols, aldehydes, and lower molecular weight acids. Organic acids are rarely present in EOs because they react with alcohols to form esters. Carotenes break down into ionones. Sulfur-containing nitrogen compounds are rare in EOs, but can be found in roasted, grilled, or roasted foods. Concretes contain high molecular weight molecules, and the presence of sulfur often imparts a very strong odor. Molecules containing sulfur and nitrogen are aglycones, glycosinolates, and isothiocyanates. Aglycones represent the sugar-free component of a glycoside, whereas glycosinolates are compounds containing sulfur or nitrogen that are derived from the combination of glycose and an amino acid. Alongside isothiocyanates, cyanates, and nitric compounds, essential oils (EOs) may contain other nitrogen-containing molecules as well [40,41].

### 1.4. Anti-Cancer Properties of Essential Oils

According to the International Agency for Research on Cancer (IARC), in 2012, the number of new cases of cancer worldwide was 14.1 million, resulting in 8.2 million deaths [42]. Currently, cancer is the leading cause of death, and it is expected to increase by 70% over the next two decades, cancers of the lung, liver, stomach, colorectal, breast, prostate and esophagus being responsible for the majority of deaths [42,43]. These statistics emphasize the increasing demand for the development of novel and innovative chemotherapeutic drugs in the coming years.

Cancer can be broadly divided into three distinct stages. First, there is the initiation stage, in which exposure to carcinogens and impaired DNA repair mechanisms leads to damage and mutations in cells. Subsequently, the promotion stage ensues, characterized by excessive cell proliferation, alterations in tissue structure, and inflammation resulting from the expansion of the initially affected cells. Finally, there is the progression stage, in which preneoplastic cells form tumors through clonal expansion, promoted by increased genomic instability and alterations in gene expression [44].

Due to the progressive nature of cancer and changes in susceptibility to treatment, each stage of carcinogenesis requires specific chemotherapeutic approaches. More specifically, tumor progression is associated with genomic instability resulting from the accumulation of mutations affecting factors involved in cell proliferation, apoptosis and DNA repair, among other processes [44,45]. Chemotherapy drugs act mainly during the promotion stage, by inhibiting cell proliferation, increasing the rate of cell death, and inducing tumor cell differentiation [46].

Although research into the use of essential oils (EOs) as cancer therapeutic agents is relatively recent, it is interesting to note that nearly half of conventional chemotherapy agents are of plant origin, of which approximately 25% are directly derived from plants and 25% are chemically modified versions of plant products [47]. An example of such molecules is paclitaxel, also known by the trade name Taxol, which was originally extracted from the bark of the *Taxus brevifolia* tree [4]. The mechanism of action of this substance relies on disrupting the process of cell division, known as mitosis, by specifically targeting tubulin, a protein component of the cellular cytoskeleton. This action triggers the activation of the mitosis checkpoint and subsequently induces apoptosis, or programmed cell death, in cancer cells. [48]. Paclitaxel is used as a therapeutic agent, alone or in combination with other drugs, to treat different types of cancer, including ovarian, breast, and pancreatic cancers [48]. Due to the depletion of natural sources, the laboratory synthesis of this drug was necessary, mainly by a synthetic route involving patchoulol, a component of essential oils, to produce patchoulol oxide [49].

More recently, researchers including Altshuler and his team have found that the enantiomer (+)-citronellal, a major component of the essential oils of *Corymbia citriodora* and *Cymbopogon nardus*, is also an effective compound in disrupting microtubule formation, similarly to well-known microtubule-disrupting agents such as colchicine and vinblastine [50]. This discovery highlights the potential of essential oils as anti-cancer therapeutic agents and opens new perspectives for their use in the treatment of cancer. However, it should be emphasized that more research is needed to assess their effectiveness and safety, as well as to determine the best application and dosage approaches.

**Figure 2:**
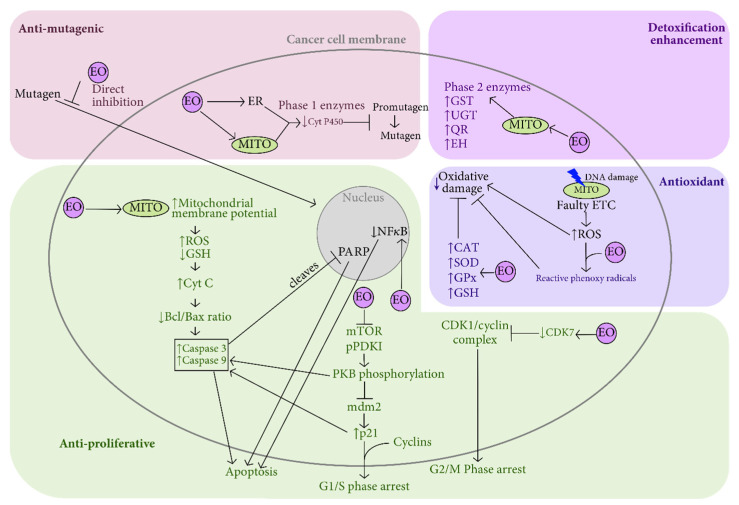
Essential oils cancer preventative and anticancer mechanisms of action. EOs possess antimutagenic, antiproliferative, antioxidant, and detoxifying capabilities acting on various pathways in the cancer cell, as well as cancer preventative capabilities. EOs may directly inhibit mutagen entry into the cell. EOs can decrease phase I enzymes such as CytC, preventing mutagen formation, and increase phase II enzymes such as GST, UGT, QR, and EH for enhanced detoxification. EOs bind ROSforming reactive phenoxy radicals, which bind more ROS and increase antioxidative enzymes CAT, SOD, GPx, and GSH, thus preventing oxidative damage as a cancer preventative mechanism. EOs disrupt mitochondrial membrane potential causing an increase in ROS and decrease in GSH, release of CytC, resulting in a cascade of disruption in Bcl/Bax ratio, increase in caspase 3 and caspase 9 activity, and PARP cleavage, resulting in apoptosis. EOs suppress mTOR and pPDK1 causing PKB dephosphorylation, which dually acts to initiate caspase activity and deactivate mdm2, causing an increase in p21 to further initiate caspase activity resulting in apoptosis. Increased p21 also induces G1/S phase cell cycle arrest. EOs cause a decrease in CDK7, blocking CDK1/cyclin complex causing G2/M phase cell cycle arrest. Bax: B-cell lymphoma 2-associated X protein; Bcl-2: B-cell lymphoma 2; CAT: catalase; CDK: cyclin-dependant kinase; CytC: cytochrome C; CytP450: cytochrome P450; EH: epoxide hydrolase; EO: essential oil; ER: endoplasmic reticulum; ETC: electron transport chain; GPx: glutathione peroxidase; GSH: glutathione; GST: glutathione S-transferase; mdm2: murine double minute 2; mTOR: mechanistic target of rapamycin; MITO: mitochondria; NFκB: nuclear factor-κB; PARP: poly ADP ribose polymerase; pPDK1: protein pyruvate dehydrogenase kinase 1; PKB: protein kinase B; QT: quinone reductase; ROS: reactive oxygen species; SOD: superoxide dismutase; UGT: uridine 5′-diphospho-glucuronosyltransferase.

EOs have demonstrated anticancer properties through various mechanisms. These include cancer prevention mechanisms, direct effects on established tumor cells, and interactions with the tumor microenvironment (Figure 2) [51,52].

### 1.5. Antitumor Properties of Essential Oils

Although significant progress has been made in comprehending the mechanisms of cell transformation, cancer continues to pose a significant global health challenge, primarily due to the emergence of multidrug resistance (MDR) in transformed cells. Cellular plasticity and flexibility, as well as high exposure to anticancer drugs, make tumors resistant [53,54,55,56]. EOs whose antitumor properties have been known since antiquity through empirical studies, have been the subject of numerous publications confirmed by in vitro studies, showing their cytotoxic action against different tumor cell lines (Table 1). Several molecules present in essential oils; in particular, phenols (such as carvacrol, thymol and eugenol), alcohols (such as linalool), and aldehydes (such as cinnamaldehyde) possess antitumor properties [57]. EOs containing high levels of these compounds typically demonstrate the most effective anti-tumor properties when tested against human cancer cell lines [58]. Certain plant essential oils, such as eucalyptus, chamomile, mugwort, and verbena officinalis, possess the ability to induce apoptosis in tumor cells. Additionally, other essential oils have the capacity to disrupt the mitochondrial membrane potential [59].

### 1.6. Antiproliferative Mechanisms of Action of Essential Oils

Resistance to cell death, sustained proliferative signaling, and evasion of growth suppressants are key hallmarks of cancer [74]. Consequently, it is vital to devise therapeutic approaches that target apoptosis (programmed cell death) induction and cell proliferation arrest. Research has shown that EOs can trigger both intrinsic (mitochondria-dependent) and extrinsic (death receptor-dependent) pathways of apoptosis.

Girola et al. (2015) examined the antitumor properties of a compound called camphene, isolated from the essential oil of *Piper cernuum*, on melanoma cells. Their results showed that this compound was able to induce apoptosis by activating the caspase-3 pathway, while also triggering endoplasmic reticulum (ER) stress signaling [75]. Another study investigated the mechanism of action of carvacrol, a monoterpenoid phenolic compound abundant in the essential oils of oregano and thyme [76]. In a metastatic breast cancer cell line called MDA-MB-231, carvacrol induced apoptosis by causing permeabilization of the mitochondrial membrane, resulting in release of cytochrome C, activation of caspases (indicated by cleavage of poly ADP ribose polymerase (PARP)), and DNA fragmentation [76]. Frankincense extracts derived from *Boswellia sacra* have also been studied and shown an ability to induce apoptosis with PARP cleavage in MDA-MB-231 cells, with increased specificity towards cancer cells [77]. Studies have also revealed that citral, present in several essential oils, induces caspase activation and, consequently, apoptosis in different types of cancer cells, including colorectal cancer and glioblastoma [78,79,80]. In addition, citral treatment was associated with reduced expression of factors promoting cancer cell growth and survival, such as aldehyde dehydrogenase 1A3 (ALDH1A3) and microtubule affinity regulatory kinase 4 (MARK4) [81,82].

Protein kinase B (PKB) is a key molecule involved in cell metabolism, transcription, cell cycle progression, and survival [83]. A study showed that *Litsea cubeba* seed oil vapor induced cell cycle arrest and apoptosis in non-small cell lung carcinoma cells, a type of cancer with a high mortality rate [84]. These effects were attributed to a significant decrease in the expression of the protein mTOR (mechanistic target of rapamycin) and phosphorylation capacity of PPDK1 (protein pyruvate dehydrogenase kinase 1), which led to the dephosphorylation of PKB and activation of the caspase-dependent apoptosis pathway [84]. In addition, PKB dephosphorylation inactivated the mdm2 (murine double minute 2) protein, leading to increased p21 expression and subsequent caspase initiation after G1 phase arrest/S of the cell cycle [84]. The dual mechanism of action of essential oils provides them with antiproliferative and antioxidant properties. Direct vapor inhalation of essential oils may offer advantages for localized delivery to the site of lung cancer [84].

In another study, Wu et al. (2013) demonstrated that administration of organosulfur compounds from garlic significantly reduced cell viability in a dose- and time-dependent manner, with diallyl trisulfide being the most effective [85]. These effects were observed in a hepatic tumor cell line called J5, where they induced G2/M phase cell cycle arrest and cell death through decreased expression of cyclin-dependent kinase (CDK) 7, resulting in inhibition of the CDK1/cyclin complex [85].

Abnormally elevated expression of nuclear factor κB (NFκB) is associated with cancer initiation and progression [86,87,88]. *α*-Terpineol, a monoterpenoid alcohol, has been shown to downregulate NFκB transcription in different tumor cell lines, with a particularly pronounced inhibitory effect on the small-cell lung carcinoma cell line NCI-H69 [89]. Additionally, *α*-terpineol has been found to have synergistic properties with linalyl acetate, another monoterpene, in colon cancer cells. This combination inhibited NFκB expression and led to apoptosis [90].

### 1.7. Cancer Cell Specificity of Essential Oils

Conventional chemotherapy drugs are more cytotoxic to cancer cells due to their higher rate of cell division. However, this cytotoxic action presents problems of cell specificity and associated toxicity for healthy cells [91]. The resulting side effects can impede healing and pose a danger to the patient’s life. Current therapeutic approaches, such as surgery followed by chemotherapy, radiotherapy, and immunotherapy offer better chances of cancer treatment and remission [91]. However, they do not fully address the need for cancer cell-specific therapy or a larger therapeutic window between normal and cancer cells. Although the new targeted strategies represent a significant improvement, they still face cell-specificity issues and high attrition when moving from preclinical studies to clinical application [91]. The use of monoclonal antibodies shows high selectivity, but limited cytotoxic activity [92]. Thus, the combined administration of monoclonal antibodies and conventional chemotherapy drugs represents a potential route to address this issue, delivering the highly cytotoxic agent specifically to cancer cells [92].

In this context, the use of essential oil (EO) extracts as unique therapeutic agents has been demonstrated in several in vitro studies, showing targeting specificity towards cancer cells and absent or greatly reduced cytotoxicity towards healthy cells, through various mechanisms of action (Table 2).

Boswellia sacra extracts have shown very promising results both in vitro and in vivo. They have been shown to be cytotoxic to three breast cancer cell lines (T47D, MCF7, and MDA-MB-231) at varying concentrations, while preserving the viability of MCF10-2A immortalized normal human breast cells [77]. This study also showed that *Boswellia sacra* extracts hydrodistilled for 12 h at 100 °C were more potent than essential oil extracts prepared at 78 °C, due to a greater amount of boswellic acid present. Apoptosis markers, such as caspase 3 activity, PARP cleavage, and DNA fragmentation, were rapidly activated in MDA-MB-231 cells, but not in MCF10-2A cells [77]. Importantly, treatment with the extracts blocked the growth of T47D-derived multicellular tumor spheroids, indicating their potential for efficacy in in vivo models [77]. Similarly, *Boswellia sacra* demonstrated cell-specific cytotoxicity in a dose-dependent manner in the bladder transitional cell carcinoma cell line J82, in contrast to the lack of cytotoxicity observed in the normal bladder cell line UROtsa [96]. Treatment of J82 cells rapidly resulted in cell shrinkage and plaque detachment, while no changes were observed in UROtsa cells. This effect was associated with a decrease in the expression of 47 genes after treatment with the essential oil extracts, whose functions include transcription factors, cell cycle regulation, and cell proliferation [96]. Finally, *Boswellia sacra* has also demonstrated cytotoxicity towards human pancreatic cells, both in culture and in a xenograft mouse model. This was manifested by repression of cell cycle regulators and activation of the caspase pathway in vitro, as well as decreased tumor cell growth and tumor cell death in vivo [101]. Similarly, similar to the findings of Suhail et al. (2011) [77], it was observed that the potency of the essential oil extract was enhanced as the hydrodistillation temperature increased. This increase in temperature positively correlated with cytotoxicity, attributed to the extraction of higher levels of boswellic acids and sesquiterpenes.

The EO extracts of *Amomum tsaoko* have demonstrated cytotoxicity against different human cancer cell lines, such as liver cancer (HepG2 and Bel-7402), cervical cancer (HeLa), gastric adenocarcinoma (SGC-7901), and prostate cancer (PC-3) [94]. It should be noted that these extracts were less effective against normal HL-7702 hepatocytes and umbilical vein endothelial cells (HUVEC) [94]. The individual components of this EO blend, namely eucalyptol and geraniol, were also evaluated [94]. Eucalyptol showed no cytotoxicity against cancer cell lines, while geraniol showed minimal cytotoxic effect on all cancer cell lines, but significantly less than the effect of the complete EO mixture [94]. Hence, the synergy between eucalyptol and geraniol, along with other components present in essential oils, may contribute to their cytotoxic activity [94].

### 1.8. Synergism of Essential Oils Extracts with Conventional Chemotherapeutic Agents: Potential of Combination Therapy Using Essential Oils

Research studies have indicated that certain specific components of essential oils can enhance the cytotoxic activity of chemotherapy drugs in various cell lines (refer to Table 3). This enhancement enables the use of lower drug concentrations while still achieving a similar therapeutic effect [102,103].

Docetaxel is commonly used as first-line treatment for hormone-refractory prostate cancer, with a median survival of approximately 20 months [102]. However, this drug is associated with serious side effects and usually administered with other treatments with dose-dependent toxicity for patients [102]. A study has shown that limonene, a specific compound, exhibits cytotoxic activity against the DU-145 prostate cancer cell line when used alone. When given in combination with docetaxel, it sensitized the cells to this drug in a dose-dependent manner, which allowed the use of much lower doses of docetaxel, reaching the IC50 in concentrations ranging from from 2.8 nM to 1.9 mM [102]. In addition, limited toxicity was observed towards normal prostate epithelial cells. Further analyses of the effects of this combination treatment revealed an increase in ROS production from mitochondrial-dependent and independent pathways, as well as an increase in cytochrome C release, p53 stabilization, and cleavage of caspase and PARP after exposure for 0 to 48 h [102].

The use of limonene not only reduces the required dosage of the toxic drug docetaxel but also offers the advantage of low toxicity in humans. Furthermore, there is a possibility that this combination could be effective in cell lines which are resistant to docetaxel [102].

*β*-Caryophyllene, although not cytotoxic as a single agent, has been shown to significantly enhance the cytotoxic activity of paclitaxel in different cancer cell lines. Specifically, the strongest effect was observed on DLD-1 cells treated with a combination of paclitaxel and 10 μg/mL *β*-caryophyllene, which resulted in an approximately 10-fold increase in paclitaxel activity [103]. *β*-Caryophyllene has been shown to increase cell membrane permeability, thereby promoting the absorption of paclitaxel. This increase in permeability is probably due to the accumulation of *β*-caryophyllene in the lipid bilayer of the membrane, which alters the permeability for substances such as paclitaxel [103].

Neutropenia is a common side effect of cancer itself, as well as treatments such as chemotherapy and radiation therapy. In particular, radiation therapy targeting sites of bone marrow proliferation can lead to neutropenia [104]. Cancer-associated neutropenia is associated with a high mortality risk due to increased susceptibility to Gram-negative bacterial infections, and when accompanied by fever, it is considered an oncology emergency [104]. Current treatment options are limited, and administration of granulocyte-colony stimulating factors (G-CSF) may be considered in some patients to promote granulocyte production in the bone marrow. In some cases, modification of the chemotherapy dose may also be appropriate [104].

A study by Zhuang et al. (2009) examined 105 patients with non-end-stage breast, colon, nasopharyngeal, or lung cancer. The results showed a significant reduction in leukocyte (14.2%) and neutrophil (11%) depletion in the treated group compared to the control group over a period of 6 weeks [105]. Flow cytometry analysis revealed a greater decrease in CD4 cells and natural killer cells in the placebo group compared with the group treated with a complex of Chinese herbal medicines (CCMH) [105]. The main component of CCMH was citronellol, a potent antioxidant compound known for its anti-cancer, anti-inflammatory, and woundhealing properties, with a concentration of 273.6 mg per capsule [105]. However, the study does not provide specific details on the role of citronellol and other components in the observed results. Therefore, the exact mechanism of action remains to be elucidated.

Previous studies have demonstrated that geraniol can increase the sensitivity of cancer cells to the conventional chemotherapeutic agent, 5-fluorouracil (5-FU), while also promoting drug absorption [106,107]. Moreover, when combined with the potent carcinogen dimethylhydrazine, geraniol demonstrated chemoprotective effects on normal colonic cells in rats [108]. This protection is explained by a reduction in DNA damage compared to control groups that did not receive essential oil extract [108].

### 1.9. Mechanisms of Anti-Cancer Action

The mechanisms of anticancer action of essential oils are multiple and complex. Some molecules present in essential oils have the ability to disrupt the cell cycle by blocking cell proliferation and inducing apoptosis (programmed cell death) [109]. Others can interfere with cell signaling and inhibit the formation of new blood vessels (angiogenesis) necessary for tumor growth [110]. In addition, essential oils can also induce oxidative damage to tumor cells by disrupting their redox balance. Some compounds also have the ability to alter the physicochemical properties of the cell membrane, which can lead to membrane instability and loss of cell function [111].

It is important to note that the mechanisms of anticancer action of essential oils can vary depending on the type of cancer targeted and specific composition of the essential oil used [112]. Additionally, more in vivo and clinical studies are essential to better understand the efficacy and safety of essential oils as a potential cancer treatment [113].

**Figure 3:**
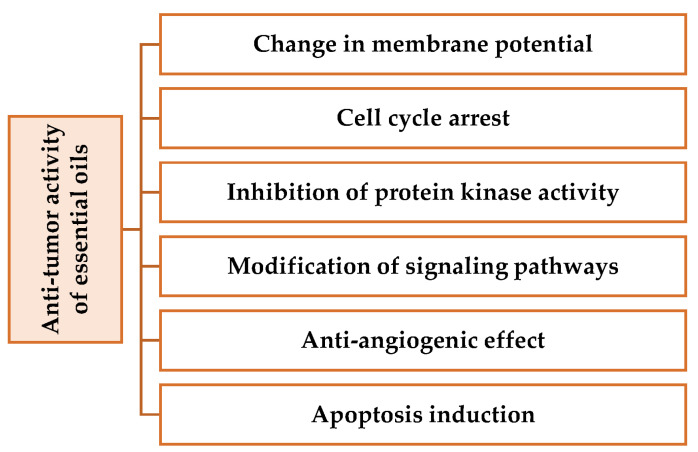
Principal mechanisms of antitumor activities for essential oils.

Although much research has been carried out in the field of chemotherapy using substances isolated from aromatic plants [114], few studies have investigated the mechanism by which whole EO or one of its constituents acts on tumor cells [115]. Although most studies report specific toxicity to cancer cells in the absence of toxicity to control cells, it is well known that some compounds such as safrole or isoeugenol are dangerous; and it is crucial to assess the toxicity of EO constituents in vitro and, in particular, in vivo [116]. The mechanisms involved are very diverse (Figure 3 and Table 4), ranging from structural levels to molecular levels (regulation of gene transcription) and metabolic levels (production of reactive oxygen species in cancer cells) [117].

### 1.10. Suppression of Inflammation and Reduction of Oxidative Stress

Inflammation and oxidative stress have been associated with the progression of cancer. However, they can also play a role in its development [138]. While multiple studies have shown that the anti-cancer properties of certain compounds found in EOs are associated with inflammation reduction, EOs also target various molecular pathways to inhibit and/or halt tumor cell proliferation. EOs can induce apoptosis by directly activating pro-apoptotic proteins or via signaling pathways, arrest the cell cycle directly or by inhibiting the activity of protein kinases, and modify the membrane potential of cancer cells [139,140].

As EOs contain several compounds, several mechanisms can act in synergy to generate an amplifying action on oxidative stress [141,142]. As an illustration, limonene has been shown to replenish diminished levels of glutathione-peroxidase, catalase, glutathione, and reductase. Similarly, eugenol has been found to restore glutathione levels in skin exposed to the carcinogen DMBA. Additionally, geraniol has demonstrated inhibitory effects on the production of nuclear factor kappa B (NF-κB), a crucial transcription factor involved in the synthesis of pro-inflammatory proteins within the body [143,144]. The connection between NF-κB’s inflammatory activity and development of cancer, resistance to therapy, tumor angiogenesis, and metastasis is extensively recognized and documented. Eugenol has been demonstrated to effectively diminish NF-κB levels in the treatment of induced gastric carcinomas in rats. Moreover, other research studies have indicated that compounds like eugenol alleviate inflammation by targeting additional factors such as cyclooxygenase-2 (COX-2), cytokines, and inflammatory molecules including IL-1β, IL-6, TNF-alpha, and PGE2. [145,146].

### 1.11. Generation of Reactive OXYGEN Species within Cancer Cells

The elevation of free radicals and oxidative stress within cancer cells can potentially exert an anti-tumor influence. Certain terpene constituents found in essential oils, like *β*-caryophyllene, demonstrate the ability to selectively stimulate the production of reactive oxygen species (ROS) within cancer cell mitochondria, while avoiding an escalation of oxidative stress in normal cells. Conversely, thymol appears to generate a stable intermediate known as a phenoxy radical, which subsequently generates free radicals and oxidized derivatives of quinones. This process has been associated with the demise of melanoma and osteosarcoma cells. [147]. Furthermore, a study conducted by Dipanwita et al. (2011) [148] demonstrated that thymol stimulates the production of hydrogen peroxide within the mitochondria of cancer cells. Additionally, eugenol has the ability to induce oxidative stress in cancer cells and reduce glutathione levels.

### 1.12. Overexpression and Detoxification of the Liver

Some terpenoids have the ability to inhibit the enzymes involved in the initiation phase of carcinogenesis. This effect is associated with the induction of phase I and phase II enzymes involved in xenobiotic metabolism. These enzymes aid in the detoxification of carcinogens, reducing their impact on DNA and thereby lowering the risk of cancer. Among these enzymes, glutathione-S-transferase is one of the most important and can be increased by 30% thanks to the action of some terpenoids, such as limonene, which is the main component of lemon EO [149,150,151].

### 1.13. Alteration of Mitochondrial Membrane Potential

An increasing body of research demonstrates the favorable impacts of essential oils on cancer, primarily through the modulation of membrane polarization in cancerous cells, especially the mitochondrial membrane. Terpenoids, due to their high lipophilicity, exhibit a notable affinity for cell membranes. Kim et al. (2012) [152], found that geraniol induces apoptosis in prostate cancer by altering the polarization of the mitochondrial membrane of cancer cells. Additionally, cancer cells are often hyperpolarized, and terpenes help depolarize the membrane, thereby restoring normal cell processes, including apoptosis [153,154]. *α*-bisabolol and thymol are also effective against acute lymphoid and myeloid leukemias by modifying the polarization of the mitochondrial membrane [155]. Germacrone appears to have a similar effect on the mitochondrial membrane of breast cancer cells, and geraniol also induces colon cell membrane depolarization [156]. *β*-elemene and other terpenes have been found to modify the membranes of cancer cells. Terpenoids can trigger the release of cofactors, including cytochrome C, from the mitochondria of cancer cells, leading to caspase-dependent apoptosis activation [157]. One pathway that can enhance the permeability of the inner mitochondrial membrane to water and small molecules is through the opening of transition pores. Terpenoids such as *α*-bisabolol seem to target this mechanism. Modulating the membrane potential can also influence the opening or closing of ion channels, subsequently altering intracellular pH and inducing various cellular responses. For instance, thymol and carvacrol are capable of inducing apoptosis through the mitochondrial pathway by opening calcium channels, leading to the release of Ca^2+^ into the endoplasmic reticulum of osteosarcoma cancer cells. [158,159].

### 1.14. Activation of Apoptosis by Caspases

Multicellular organisms use two distinct mechanisms to regulate their cells: apoptosis and necrosis. Apoptosis, also known as programmed cell death, is crucial for embryonic development, cell differentiation, maintenance of tissue homeostasis, and regulation of the immune system [160]. This complex process involves the activation of genes involved in programmed death, and mutations in these genes can lead to various human diseases, including cancer [161]. Key features of apoptosis include condensation of cytoplasm and nucleolus, DNA fragmentation, and polymerase degradation [162]. Apoptosis is a crucial pathway for anticancer agents, although tumor cell resistance to most cytostatic agents poses a major challenge in cancer therapy [163]. Understanding the signaling pathways that control the induction of apoptosis by cytostatic agents in tumor cells is therefore crucial to improve cancer therapy [164].

Numerous studies have demonstrated that specific compounds like thymol, thymoquinone, and terpenes possess the ability to induce apoptosis through both caspase-independent and caspase-dependent pathways, albeit in a tissue-specific manner [165]. These agents have exhibited their efficacy in activating various caspases, notably caspases 3, 7, 8, and 9, which are triggered in different cancer cell lines encompassing prostate, glioma, breast, colon, lung, and leukemia [166]. *β*-Caryophyllene, *α*-bisabolol and *β*-elemene are other terpenes that can activate caspases. Finally, germacrone has also been identified as an activator of caspases 3, 7 and 9 [167].

### 1.15. Cell Cycle Arrest

In a study by Yin et al. [168], it was demonstrated that thymol can impede the transition of the cell cycle from the G0 phase to the G1 phase. Similarly, Rajput et al. [169] found that thymoquinone specifically targets the Akt pathway, inhibits cyclin D1, halts the cell cycle, and induces apoptosis in breast cancer cells. Additionally, in another study, Tundis et al. (2009) [170] revealed the participation of PARPγ pathways in the anti-cancer effects of thymoquinone on breast cancers. In vitro investigations have indicated that prior exposure of tumor cells to thymoquinone, followed by the administration of gemcitabine or oxaliplatin, enhances growth inhibition compared to using gemcitabine or oxaliplatin alone. [171]. The mechanisms underlying these effects involve the dysregulation of NF-κB, Bcl-2 family genes, and NF-κB-dependent antiapoptotic genes. Thymoquinone disrupts the expression of NF-κB, which provides an explanation for its diverse cellular activities. Sethi et al. (2008) [172] also showed that this compound is involved in the activation of apoptosis pathways via the suppression of NF-κB. In humans, thymoquinone inhibits cell proliferation in melanomas, sensitizes to chemotherapy, and activates STAT transcription pathways. In another study conducted by Yazan et al. (2009) [173], it is proposed that this compound exhibits a cytotoxic effect by inducing apoptosis through a P53-dependent signaling pathway.

### 1.16. Modification of Signaling Pathways (Disabling the PI3K/Akt/NF-κB Pathway)

In order to control cancer growth, deactivating the PI3K/Akt/NF-κB pathway has been identified as a strategic approach [174]. Numerous cancers, including leukemia, exhibit overexpression of genes that lead to phosphoinositide 3-kinase (PI3K)/Akt activation, making it a crucial target for cancer therapy [175]. The PI3K signaling pathway plays a role in regulating cell growth and glucose metabolism [176]. It impacts protein synthesis through the mTOR enzyme and influences glucose uptake and utilization [177]. Activation of the PI3K pathway renders cancer cells dependent on high glucose flux [178]. By targeting this pathway, compounds like geraniol can influence the metabolic aspect of cancers, such as prostate cancer that heavily relies on glucose for energy production [179]. Cancer cells predominantly rely on aerobic glycolysis in the cytoplasm (fermentation of pyruvate to lactate, resulting in only two ATP molecules per glucose molecule) rather than oxidative phosphorylation in the mitochondria (production of 36 ATP molecules per glucose molecule) [180]. Geraniol’s inhibition of mTOR in cancer cells can also reactivate a process known as “autophagy,” leading to cell death [181]. Derivatives of *β*-Caryophyllene have also shown potential in acting through this biochemical pathway against breast and prostate cancers, while *α*-bisabolol demonstrates activity against pancreatic cancer and *β*-elemene exhibits effectiveness against stomach and lung cancers [182].

### 1.17. Modification of the AMPK Pathway

AMP-activated protein kinase (AMPK) serves as a cellular energy state sensor and plays a crucial role in regulating energy metabolism [183]. While its beneficial effects have primarily been associated with type II diabetes, there is increasing interest in its involvement in cancer cells [184]. This protein functions as a mediator for the tumor suppressor LKB1 and, when stimulated, it reprograms cellular metabolism and influences the p53 biochemical pathway, which plays a role in the series of events leading to caspase activation and apoptosis in cancer cells [185]. Some studies indicate that terpenoids like geraniol and *β*-caryophyllene can activate AMPK, leading to the inhibition of cell growth and apoptosis in bladder, prostate, and breast cancers [186].

### 1.18. Modulation in the Expression of MAPK/ERK Proteins

MAPK/ERK proteins are members of a family of proteins present in cells that transmit signals from membrane receptors to DNA at the level of the nucleus. Signaling begins when a signaling molecule binds to a receptor on the cell membrane, followed by signal transduction and then signaling pathways that culminate in the production of a second messenger [187]. The second information mentioned activates DNA within the nucleus, leading to protein expression and consequent cellular changes, including cell division. MAPK/ERK proteins, which are kinases, operate by adding phosphate groups to nearby proteins, triggering a phosphorylation-dephosphorylation reaction that acts as an on/off switch. Certain compounds like limonene and *β*-elemene appear to function similarly by promoting apoptosis in cancer cells, particularly in lymphomas [188].

### 1.19. Inhibition of the Activity of 3-Hydroxy-3-Methylglutaryl-Coenzyme A

The enzyme 3-hydroxy-3-methylglutaryl-coenzyme a reductase is essential in cholesterol metabolism because it catalyzes the formation of mevalonate, a precursor necessary for cell proliferation. Depletion of mevalonates results in arrest in the G1 phase of the cell cycle. Thus, inhibiting mevalonate synthesis may be an effective strategy to limit cancer cell growth. Some terpenoids, such as farnesol, have the ability to inhibit this synthesis. Indeed, studies have shown that farnesol may have this effect on liver cancer in rats. *β*-ionone also showed similar properties by reducing cholesterol synthesis [189,190].

### 1.20. Anti-Angiogenic Effect

Tumors rely on the nutrients supplied by blood capillaries through a process called angiogenesis. However, certain compounds found in EOs, such as terpenes and certain polyphenols, can hinder the formation of vascular networks that nourish tumors, as exemplified by limonene. In a recent study conducted by Chen et al. (1998) [130], it was observed that the group treated with limonene exhibited a significantly lower microvessel density (5.32 ± 4.26) compared with the control group (18.64 ± 2.81). Furthermore, the expression of vascular endothelial growth factor (VEGF) was also markedly reduced in the limonene group (29.71 ± 8.92 vs. 45.77 ± 4.79). Similarly, *β*-elemene has demonstrated effective reduction of VEGF both in laboratory studies and in live organisms. Another study revealed that administering 100 mg/kg of eugenol (three times a week) induced apoptosis in tumor cells and significantly decreased VEGF and matrix metalloproteinase, providing clear evidence of its antiangiogenic effect [191,192,193].

### 1.21. Modification of Histones

Histones are essential proteins that coil around DNA, creating nucleosomes as structural units. The positively charged nature of histones enables them to strongly interact with the negatively charged phosphate groups present in DNA. This interaction plays a critical role in DNA packaging and organization. Post-translational modifications of histones, such as methylation, phosphorylation, acetylation, ubiquitination, and acylation, impact the chromatin state and, therefore, expression regulation gene. Recent studies have indicated that the inhibition of cancer cell growth by specific terpenes, including *β*-elemene, may be associated with an elevation in histone H1 levels. Histone H1 is recognized as a transcription inhibitor, suggesting that the increased abundance of this histone could contribute to the observed suppression of cancer cell proliferation [194,195,196].

### 1.22. Inhibition of Other Factors Involved in the Induction of Tumorigenesis

Essential oils (EOs) contain monoterpenoids that inhibit isoprenylation, a chemical reaction carried out by enzymes like the protein farnesoltransferase, on some proteins in cells. These prenylated proteins activate promoters of genes involved in cell growth and proliferation [197]. Monoterpenoids disrupt these reactions by competing with them, thereby slowing down or obstructing the activities of cell signaling proteins that promote cancer cell growth [198]. For instance, compounds like limonene or perillylic acid reduce the levels of growth factors such as mitogen IGF-II while increasing the presence of stabilizing factors like TGF-β. These effects have been observed in mammary tumor cells, where they induce cell cycle arrest in the G1 phase, ultimately leading to the death of tumor cells [199]. EOs inhibit the Ras family protein pathway and related genes, which have a crucial role in promoting cancer growth by accelerating cancer cell division [200]. Limonene, for example, blocks this pathway and thus prevents the development of cancer. In addition, terpenoids have an effect on hypoxia-inducible factor-1α (HIF)-1α, which actively participates in human cancerous tumors. Studies show that *β*-elemene, for example, significantly inhibits the expression of these proteins [201].

### 1.23. Routes of Administration of Essential Oils

In general, there are four main interfaces that connect the body to the outside world: digestive, cutaneous, pulmonary, and genito-urinary [202]. Currently, all forms of injections (intramuscular, intravenous, subcutaneous) are contraindicated for essential oils (EO) due to the knowledge and galenic forms available. EOs are aromatic molecules whose absorption depends on their mode of administration [203]. The oral (or per os) route is the first considered and allows digestive absorption of essential oils at all levels of the digestive tract. This absorption occurs mainly by passive diffusion through the lipid bilayer, but some aromatic molecules can also undergo active transport targeting levorotatory rather than dextrorotatory isomers [204]. The respiratory tract is another efficient route of administration for EOs, avoiding intestinal and hepatic first-pass metabolism and allowing rapid absorption into the general circulation [205]. Terpenes, which are readily metabolized by oxidation, hydroxylation, and conversion to glucuronides or sulfates, are typically absorbed by the body via oral, transdermal, or inhalation routes [206,207]. Elimination occurs rapidly through faeces and urine, often within one to three days. EO compounds are transported by the blood and can act either in their native form or in the form of derived metabolites [208]. Studies have shown that the glucuronides and sulphates derived from carvacrol and thymol (main components of the HE of *Origanum compactum*) are quickly found in the urine after oral absorption in rats [209]. Limonene, administered orally in rats and man, is also completely absorbed from the intestine, with almost total elimination in the urine in the form of derived metabolites [210]. EO compounds can also be absorbed through the skin, as shown in a study of carvone enantiomers, which were better absorbed when applied to the skin. The two isomers tested ([R] and [S] isomers) are not metabolized by the same pathway [211].

### 1.24. Toxicity of Essential Oils

In general, oils and aromatic substances used under medical supervision and in physiological doses do not show toxicity. However, toxic problems can arise from confusion, suicide attempts, or irresponsible self-medication. Some aromatic molecules are potentially very toxic and are therefore prohibited or restricted in the pharmaceutical industry, perfumery, food flavorings, and nutraceuticals [212,213]. For example, menthol, which is the main component of peppermint essential oil [*Mentha x piperita* (L) var. piperita] and Japanese mint [*Mentha arvensis* (L) var. piperascens] can be very toxic when given in high doses, causing abdominal pain, nausea, vomiting, dizziness, ataxia, convulsions, drowsiness, and then coma. Products containing menthol are not recommended for children under 30 months, regardless of their mode of administration. In addition, some diseases, such as glucose-6-phosphate dehydrogenase deficiency (favism), increase the toxicity of menthol [214].

Thujone, a monoterpene ketone present in the essential oils of Artemisia absinthium, Thuja occidentalis, Salvia officinalis, Artemisia vulgaris, Artemisia afra, Artemisia arborescens and Artemisia herba-alba, exists in two isomeric forms, α and β, the α form being the most toxic. *α*-Thujone is a GABA-A receptor antagonist and has stimulant and convulsant effects. It has also been suggested that thujone reacts with the same receptors as tetrahydrocannabinol. Thujone poisoning can cause epileptic seizures, cyanosis, intermittent hypotonia, hyporeflexia, and loss of consciousness [215].

Pulegone, a compound found in the essential oils of pennyroyal from *Mentha pulegium*, *Hedeoma pulegioides*, *Agasthoma betulina*, and *Mentha arvensis*, is another potentially toxic aromatic substance. EOs rich in pulegone can cause hepatocellular necrosis leading to sometimes fulminant acute hepatitis. The mechanism of toxicity is beginning to be better understood: pulegone, by transforming into menthofuran and various electrophilic metabolites of menthofuran, can covalently bind to CYP2A6 and NADPH-P450-reductase proteins and reduce, in a time-dependent manner and the concentration, the activity of human CYP2A6. These two effects may partly explain the hepatocellular damage caused by these compounds [216].

## 2. Conclusions

The ability of EOs to inhibit cancer cell growth without affecting healthy cells is linked to their ability to activate specific molecular targets that induce cell death. Numerous studies have revealed that some constituents of EOs, such as carvacrol, can be considered a new class of anticancer drugs that are highly effective in shrinking tumors while exhibiting low toxicity. Additionally, several studies have explored the synergistic effects of EOs with other compounds, including conventional drugs, but these studies are limited to in vitro conditions. In this type of experiment, it is difficult to understand the effects obtained, because EOs are complex mixtures of molecules with very varied chemical structures. Therefore, the action of EOs on cancer cells is the result of the effect of each individual compound modulated by the potential action of the synergistic effect. Despite these challenges, it is important to encourage studies on the synergistic effects of EOs with conventional chemotherapy, as these mixtures may provide promising sources of new anti-cancer agents. Exploring the anticancer properties of EOs and their active components is a current research area that needs to be studied in parallel with conventional chemotherapy.

## 3. Future Perspectives

Future research should focus on investigating the synergistic effects of EOs with conventional drugs, although these studies are currently limited to in vitro conditions. Understanding the potential interactions between EOs and standard chemotherapy agents could lead to the development of new and more effective anti-cancer treatments. Exploring the active components of EOs and their mechanisms of action is an important area of ongoing research. By studying EOs and their compounds in parallel with conventional chemotherapy, researchers may discover new sources of anti-cancer agents and potentially enhance the efficacy of existing treatments.

In summary, further studies are needed to unravel the complexities of EOs and their interactions with cancer cells. The exploration of synergistic effects and identification of active compounds within EOs could open new avenues for developing innovative anti-cancer therapies.

## Figures and Tables

**Table 1:** Essential oils composition of some plants with antitumor properties.

Family	Main Components	References
Cupressaceae	*β*-pinene; *α*-pinene.	[60]
Cupressaceae	D-verbenol; Cedrol; L-verbenol.	[61]
Cupressaceae	Camphor; Bornyl d’acetate.	[62]
Euphorbiaceae	Viridiflorene.	[63]
Flacourtiaceae	*β*-caryophyllene; *α*-humulene.	[64]
Lamiaceae	1,8-cineole;	[65,66,67,68]
	Borneol;	
	Camphor;	
	Carvacrol;	
	D-germacrene;	
	Limonene;	
	p-cymene;	
	Thymol;	
	*α*-thujone;	
	γ-terpinene.	
Meliaceae	Caryophyllene; *β*-caryophyllene.	[69]
Myricaceae	*β*-caryophyllene; *α*-humulene.	[70]
Myrtaceae	*α*-pinene; *β*-caryophyllene.	[71]
Rosaceae	*α*-pinene; Sabinene; γ-eudesmol; *α*-thujene; *α*-humulene.	[72]
Rutaceae	*β*-caryophyllene; *β*-elemene.	[73]

**Table 2:** Essential oils bearing plants and major constituents with targeted cytotoxicity to cancer cells in in vitro studies.

Species	Major EO Constituent(s)	Cancer Cell Lines	Noncancer Cell Lines	Major Findings and EO Concentrations	Mechanisms	Reference
*Thymus fallax*	Carvacrol, p-cymene, thymol, *γ*-terpinene	DLD-1 (CRc)	Mouse fibroblast (L.929)	Cytotoxic to cancer cells (IC_50_ 0.347 mg/mL) and noncytotoxic to normal cells (IC_50_ 22 mg/mL)	Antioxidant activity	[93]
*Boswellia sacra*	*α*-pinene, *α*-thujene, *β*-pinene, myrcene, boswellic acid	T47D, MCF7, MDA-MB-231 (Bc)	Immortalized normal human breast (MCF10-2A)	Cytotoxic to cancer cells (EO dilution IC_50_ 1:900 for TD47, 1:1000 for MCF7, 1:950 for MDA-MB-231) and noncytotoxic to immortalized normal cells (EO dilution IC_50_ 1:680)	Antiproliferative	[77]
*Amomum tsaoko*	1,8-cineole, *ρ*-propylbenzaldehyde, geraniol, geranial, *α*-terpineol, *α*-phellandrene, neral, *β*-pinene	HepG2 and Bel-7402 (Lc) HeLa (Cc), A549 (Lc), SGC-7901 (GAC), PC-3 (Pc)	Hepatocyte (HL-7702) and umbilical vein endothelial (HUVEC)	Cytotoxic to cancer cells, particularly HepG2 (IC_50_ 31.8 μg/mL), Hela (IC_50_ 66.46 μg/mL) and Bel-7402 (IC_50_ 96.08 μg/mL), with less cytotoxicity towards HL-7702 (IC_50_ 272.4 μg/mL) and HUVEC (IC_50_ 163.91 μg/mL). No cytotoxicity towards A549	Antiproliferative	[94]
*Lippia alba* (Citral chemotype)	Geranial, neral, geraniol, *trans*-*β*-caryophyllene, 6-methyl-5-hepten-2-one, limonene, linalool	HeLa (Cc)	African green monkey kidney (Vero)	Cytotoxic to cancer cells (CC_50_ 3.5 μg/mL) and noncytotoxic to normal cells (CC_50_ > 100 μg/mL)	Citral-dependent cytotoxicity	[95]
*Boswellia* sp. (1200 mg/mL frankincense gum resin)	Duva-3,9,13-trien-1,5alpha-diol-1-acetate, octyl acetate, o-methyl anisole, naphthalene decahydro-1,1,4a-trimethyl-6-methylene-5-(3-methyl-2-pentenyl), thunbergol (Mikhaeil et al., 2003)	J82 (Blc)	Human urothelium (UROtsa)	Cytotoxic to cancer cells (no viable cells after EO dilution 1:1100 after 24 h) and noncytotoxic to normal cells (no viable cells after EO dilution 1:400)	Antiproliferative	[96]
*Casearia sylvestris*	Bicyclogermacrene, *β*-caryophyllene, spathulenol, *α*-humulene, *α*-pinene	HeLa (Cc), A549 (Lc) HT-29 (CRc)	Monkey kidney (Vero) and mice macrophages	Cytotoxic to HeLa (CD_50_ 63.3 µg·ml^−1^), A549 (CD_50_ 60.7 µg·ml^−1^) and HT-29 (CD_50_ 90.6 µg·ml^−1^) with less cytotoxicity to Vero (CD_50_ 210.1 µg·ml^−1^) and macrophages (CD_50_ 234.0 µg·ml^−1^)	Cytotoxicity	[97]
*Zanthoxylum rhoifolum* Lam	ß-caryophyllene, *α*-humulene, *α*-pinene, myrcene, linalool	HeLa (Cc), A549 (Lc) HT-29 (CRc)	Monkey kidney (Vero) and mice macrophages	Cytotoxic to HeLa (CD_50_ 90.7 µg/mL), A549 (CD_50_ 82.3 µg/mL), and HT-29 (CD_50_ 113.6 µg/mL) and noncytotoxic to normal cells (CD_50_ > 600 µg/mL)	Cytotoxicity	[98]
*Commiphora gileadensis*	Sabinene, ß-caryophyllene, germacrene D, *α*-pinene	BS-241 (Mouse T-cell lymphoma) MoFir (Epstein Barr virus transformed human B lymphocytes)	Normal human skin fibroblasts (FB)	EO dilution of 1:5000 killed 87% of BS-24-1 cells and 40% of MoFir cells	Antiproliferative	[99]
*Aniba rosaeodora*	Rosewood essential oil (REO), linalool	A431 (Ec), HaCaT (pre-cancerous)	Epidermal keratinocytes (HEK001, NHEK)	Cytotoxicity to cancer cells A431 and HaCaT (<20% viability) and minor cytotoxicity to normal cells HEK001 and NHEK (>70% viability)	Cytotoxicity	[100]

Note. Cytotoxicity is expressed as the concentration of the essential oils inhibiting cell growth by 50%; CRc: colorectal cancer; Bc: breast cancer; Lc: lung cancer; Cc: Cervical cancer; GAC: gastric adenocarcinoma; Pc: prostate cancer; BLc: bladder carcinoma; Ec: epidermoid carcinoma; IC50: inhibitor concentration 50; CC50: cytotoxic concentration.

**Table 3:** In vitro studies of essential oils in combination with conventional chemotherapy agents.

Cell Lines	Chemotherapy Drug Used Alone and Concentration	EO Constituent Used Alone and Concentration	Combined EO and Chemotherapy Drug		Reference
Prostate cancer cell (DU-145)	Docetaxel IC_50_ 2.8 nM	*d*-limonene IC_50_ 2.8 mM	IC_50_ docetaxel 1.9 mM and d-limonene 0.2 mM		[102]
Human breast cancer (MCF-7)	Paclitaxel 0.025 µg/mL^−1^ resulted in 28% cell growth inhibition	*β*-caryophyllene resulted in no inhibition of cell growth	*β*-caryophyllene 2.5 µg/mL^−1^ and Paclitaxel 0.025 µg/mL^−1^ resulted in 50% cell growth inhibition	*β*-caryophyllene 10 µg/mL^−1^ and Paclitaxel 0.025 µg/mL^−1^ resulted in 68% cell growth inhibition	[103]
Human colorectal adenocarcinoma (DLD-1)	Paclitaxel 0.025 µg/mL^−1^ resulted in 17.3% cell growth inhibition	*β*-caryophyllene resulted in no inhibition of cell growth	*β*-caryophyllene 2.5 µg/mL^−1^ and Paclitaxel 0.025 µg/mL^−1^ resulted in 91% cell growth inhibition	*β*-caryophyllene 10 µg/mL^−1^ and Paclitaxel 0.025 µg/mL^−1^ resulted in 189% cell growth inhibition	[103]
Mouse fibroblast (L-929)	Paclitaxel 0.025 µg/mL^−1^ resulted in 18.4% cell growth inhibition	*β*-caryophyllene resulted in no inhibition of cell growth	*β*-caryophyllene 2.5 µg/mL^−1^ and Paclitaxel 0.025 µg/mL^−1^ resulted in 36% cell growth inhibition	*β*-caryophyllene 10 µg/mL^−1^ and Paclitaxel 0.025 µg/mL^−1^ resulted in 123% cell growth inhibition	[103]

**Table 4:** Antitumor activity of the main components of essential oils.

Components	Antitumor Activity	Animal and/or Cell Line Tested	References
Carvacrol	✓ The halting of cell cycle progression and the initiation of apoptosis ✓ Antioxidant activity ✓ Inhibition of DNA synthesis ✓ Prevention of hepatocellular carcinogenesis	✓ Human breast adenocarcinoma (MCF-7) ✓ Leukemic cell line in mice ✓ Malignant colon cell line (Caco-2) ✓ Induced tumor in rats ✓ The cell line used in this study is HepG2, derived from hepatocellular adenocarcinoma	[118,119,120,121,122]
Geraniol	✓ Cell cycle arrest ✓ Morphological and functional blocking of cell differentiation ✓ The impact of geraniol on the metabolism of fatty acids and mevalonate in human cell lines ✓ Apoptosis induction and inhibition of RhoA activation	✓ The colon cancer cell line used in this study is Caco-2 ✓ Human cancer cell colonies ✓ The cell line employed in this study is HepG2, which represents human hepatocellular carcinoma. Additionally, the study explores hepatocarcinogenesis in rats	[123,124,125,126]
Limonene	✓ Stimulation of apoptosis and the exertion of an antiangiogenic impact ✓ Activation of detoxification enzymes such as glutathione-S-transferase (GST) and uridine diphosphate glucuronosyltransferase (UDPGT) ✓ Strong affinity with HMG-CoA reductase ✓ Effects on the intracellular communication gap junction	✓ Adenocarcinoma of human colorectal cancer ✓ Induction of carcinogenesis in rat mammary (DMBA) ✓ In silico approaches ✓ Human pancreatic carcinoma cells (PaCa)	[127,128,129,130]
Linalool	✓ Elevation of reactive oxygen species (ROS) and reduction in ATP levels ✓ Induction of apoptosis via activation of p53 and CDKIs	✓ Hepatocellular carcinoma (HepG2) ✓ Human lymphoma (Raji)	[131,132]
Thymol	✓ Halting of the cell cycle progression and initiation of apoptosis ✓ Antioxidant activity	✓ Human breast adenocarcinoma (MCF-7) ✓ Melanoma lineage (B16-F10) ✓ Mast Cell Cell Line (P815) ✓ Human intestinal cell line (Caco-2) ✓ Human hepatocellular (HepG2)	[133,134,135]
Thymoquinone	✓ Suppression of Akt phosphorylation, promotion of apoptosis, and inhibition of HDAC2 protein ✓ Stimulation of apoptosis ✓ Halting of the cell cycle progression and induction of apoptosis by influencing the Akt pathway ✓ Regulation of the activation of the PPAR-γ pathway	✓ Melanoma cell line (51L8A) ✓ Human mammary adenocarcinoma (MDA-MB-468) ✓ Breast cancer cell line (MDA-MB-231)	[136,137]

## Data Availability

Data is contained within the article.

## References

[1] Cammarota G., Ianiro G., Ahern A., Carbone C., Temko A., Claesson M.J., Gasbarrini A., Tortora G. (2020). Gut microbiome, big data and machine learning to promote precision medicine for cancer. Nat. Rev. Gastroenterol. Hepatol..

[2] Kushi L.H., Doyle C., McCullough M., Rock C.L., Demark-Wahnefried W., Bandera E.V., Gapstur S., Patel A.V., Andrews K., Gansler T. (2012). American Cancer Society Guidelines on Nutrition and Physical Activity for cancer prevention: Reducing the risk of cancer with healthy food choices and physical activity. CA Cancer J. Clin..

[3] Czarnecka A.M., Bartnik E., Fiedorowicz M., Rutkowski P. (2020). Targeted therapy in melanoma and mechanisms of resistance. Int. J. Mol. Sci..

[4] Cristini V., Lowengrub J. (2010). Multiscale Modeling of Cancer: An Integrated Experimental and Mathematical Modeling Approach.

[5] Ladeiro Y., Couchy G., Balabaud C., Bioulac-Sage P., Pelletier L., Rebouissou S., Zucman-Rossi J. (2008). MicroRNA profiling in hepatocellular tumors is associated with clinical features and oncogene/tumor suppressor gene mutations. Hepatology.

[6] World Health Organization (2008). WHO Report on the Global Tobacco Epidemic, 2008: The MPOWER Package.

[7] Cao W., Chen H.D., Yu Y.W., Li N., Chen W.Q. (2021). Changing profiles of cancer burden worldwide and in China: A secondary analysis of the global cancer statistics 2020. Chin. Med. J..

[8] Bernardes N., Chakrabarty A.M., Fialho A.M. (2013). Engineering of bacterial strains and their products for cancer therapy. Appl. Microbiol. Biotechnol..

[9] Cragg G.M., Grothaus P.G., Newman D.J. (2009). Impact of natural products on developing new anti-cancer agents. Chem. Rev..

[10] Abdoul-Latif F.M., Oumaskour K., Boujaber N., Ainane A., Mohamed J., Ainane T. (2021). Formulations of a cosmetic product for hair care based on extract of the microalga Isochrysis galbana: In vivo and in vitro activities. J. Anal. Sci. Appl. Biotechnol..

[11] Hewlings S.J., Kalman D.S. (2017). Curcumin: A review of its effects on human health. Foods.

[12] Balandrin M.F., Kinghorn A.D., Farnsworth N.R. (1993). Plant-derived natural products in drug discovery and development: An overview. Human Medicinal Agents from Plants.

[13] Mohamed Abdoul-Latif F., El Montassir Z., Ainane A., Gharby S., Sakar E.H., Merito A., Mohamed J., Ainane T. (2022). Use of Thymus Plants as an Ecological Filler in Urea-Formaldehyde Adhesives Intended for Bonding Plywood. Processes.

[14] Al-Radadi N.S. (2022). Biogenic proficient synthesis of (Au-NPs) via aqueous extract of Red Dragon Pulp and seed oil: Characterization, antioxidant, cytotoxic properties, anti-diabetic anti-inflammatory, anti-Alzheimer and their anti-proliferative potential against cancer cell lines. Saudi J. Biol. Sci..

[15] Majdalawieh A.F., Fayyad M.W., Nasrallah G.K. (2017). Anti-cancer properties and mechanisms of action of thymoquinone, the major active ingredient of Nigella sativa. Crit. Rev. Food Sci. Nutr..

[16] Bhavaniramya S., Vishnupriya S., Al-Aboody M.S., Vijayakumar R., Baskaran D. (2019). Role of essential oils in food safety: Antimicrobial and antioxidant applications. Grain Oil Sci. Technol..

[17] Rios J.L. (2016). Essential oils: What they are and how the terms are used and defined. Essential Oils in Food Preservation, Flavor and Safety.

[18] Alam M., Kamal A., Upadhyay T.K., Upadhye V.J. (2010). The role and effects of aroma: Status and trends. Aromatic Plants: The Technology, Human Welfare and Beyond.

[19] Khammour F., Abdoul-Latif F.M., Ainane A., Mohamed J., Ainane T. (2021). Eco-friendly adsorbent from waste of mint: Application for the removal of hexavalent chromium. J. Chem..

[20] Mukhija M., Sundriyal A., Loshali A. (2022). Essential Oils and Their Biological Applications: Extraction methods, Types, Biological Activities, Antimicrobial Fumes. Handbook of Research on Advanced Phytochemicals and Plant-Based Drug Discovery.

[21] Butnariu M. (2021). Plants as source of essential oils and perfumery applications. Bioprospecting of Plant Biodiversity for Industrial Molecules.

[22] Rehman R., Hanif M.A., Mushtaq Z., Al-Sadi A.M. (2016). Biosynthesis of essential oils in aromatic plants: A review. Food Rev. Int..

[23] Atemni I., Touijer H., Hjouji K., Tlemcani S., Ainane T., Taleb M., Rais Z. (2023). Effect of bone meal on growth traits, photosynthetic pigment content, and essential oil chemical composition of *Pelargonium graveolens*. Ind. Crops Prod..

[24] Zeroual A., Sakar E.H., Ibourki M., Bijla L., Ainane A., Mahjoubi F., Chaouch M., Gharby S., Chaqroune A., Ainane T. (2021). Phytochemical screening and mineral profiling of wild and cultivated rosemary (*Rosmarinus officinalis* L.) from Taounate region (northern Morocco). Pharmacol. Online.

[25] Cseke L.J., Kaufman P.B., Kirakosyan A. (2007). The biology of essential oils in the pollination of flowers. Nat. Prod. Commun..

[26] Sharmeen J.B., Mahomoodally F.M., Zengin G., Maggi F. (2021). Essential oils as natural sources of fragrance compounds for cosmetics and cosmeceuticals. Molecules.

[27] Ainane T., Elkouali M.H., Ainane A., Talbi M. (2014). Moroccan traditional fragrance based essential oils: Preparation, composition and chemical identification. Der Pharma Chem..

[28] Bilia A.R., Guccione C., Isacchi B., Righeschi C., Firenzuoli F., Bergonzi M.C. (2014). Essential oils loaded in nanosystems: A developing strategy for a successful therapeutic approach. Evid. -Based Complement. Altern. Med..

[29] Abdoul-latif F.M., Ainane A., Abdoul-latif T.M., Ainane T. (2020). Chemical study and evaluation of insectical properties of African Lippia citriodora essential oil. J. Biopestic..

[30] Ignea C., Raadam M.H., Koutsaviti A., Zhao Y., Duan Y.T., Harizani M., Miettinen K., Georgantea P., Rosenfeldt M., Viejo-Ledesma S.E. (2022). Expanding the terpene biosynthetic code with non-canonical 16 carbon atom building blocks. Nat. Commun..

[31] Hillier S.G., Lathe R. (2019). Terpenes, hormones and life: Isoprene rule revisited. J. Endocrinol..

[32] Chang T.H., Hsieh F.L., Ko T.P., Teng K.H., Liang P.H., Wang A.H.J. (2010). Structure of a heterotetrameric geranyl pyrophosphate synthase from mint (*Mentha piperita*) reveals intersubunit regulation. Plant Cell.

[33] Barja Afonso M. (2019). Exploring the Diversity of Geranylgeranyl Diphosphate Synthases in Arabidopsis thaliana and Solanum lycopersicum.

[34] Abdoul-Latif F.M., Ejjabraoui M., Ainane A., Hachi T., Mohamed J., Oumaskour K., Boujaber N., El Montassir Z., Ainane T. (2023). Correlation of the Diffusion Parameters and the Biological Activities in the Formulation of *Pinus halepensis* Essential Oil in Phosphogypsum Material. Appl. Sci..

[35] Stephane F.F.Y., Jules B.K.J. (2020). Terpenoids as important bioactive constituents of essential oils. Essential Oils-Bioactive Compounds, New Perspectives and Applications.

[36] Sadgrove N.J., Padilla-González G.F., Phumthum M. (2022). Fundamental chemistry of essential oils and volatile organic compounds, methods of analysis and authentication. Plants.

[37] Vassão D.G., Davin L.B., Lewis N.G. (2008). Metabolic engineering of plant allyl/propenyl phenol and lignin pathways: Future potential for biofuels/bioenergy, polymer intermediates, and specialty chemicals?. Adv. Plant Biochem. Mol. Biol..

[38] Mohamed Abdoul-Latif F., Ainane A., Houmed Aboubaker I., Merito Ali A., El Montassir Z., Kciuk M., Mohamed J., Ainane T. (2023). Chemical Composition of the Essential Oil of *Catha edulis* Forsk from Djibouti and Its Toxicological Investigations In Vivo and In Vitro. Processes.

[39] Dhifi W., Bellili S., Jazi S., Bahloul N., Mnif W. (2016). Essential oils’ chemical characterization and investigation of some biological activities: A critical review. Medicines.

[40] Pengelly A. (2021). The Constituents of Medicinal Plants.

[41] Bhatla S.C., Lal A.M., Bhatla S.C. (2018). Secondary metabolites. Plant Physiology Development and Metabolism.

[42] World Health Organization (WHO) (2019). Internacional Agency for Research on Cancer. Cancer Fact Sheets.

[43] World Health Organization (2015). Cancer.

[44] Ferguson L.R., Chen H., Collins A.R., Connell M., Damia G., Dasgupta S., Malhotra M., Meeker A.K., Amedei A., Amin A. (2015). Genomic instability in human cancer: Molecular insights and opportunities for therapeutic attack and prevention through diet and nutrition. Seminars in Cancer Biology.

[45] Fresco P., Borges F.I.G.M., Diniz C., Marques M.P.M. (2006). New insights on the anticancer properties of dietary polyphenols. Med. Res. Rev..

[46] Morse M.A., Stoner G.D. (1993). Cancer chemoprevention: Principles and prospects. Carcinogenesis.

[47] Amin A., Gali-Muhtasib H., Ocker M., Schneider-Stock R. (2009). Overview of major classes of plant-derived anticancer drugs. Int. J. Biomed. Sci. IJBS.

[48] Weaver B.A. (2014). How Taxol/paclitaxel kills cancer cells. Mol. Biol. Cell.

[49] Holton R.A., Kim H.B., Somoza C., Liang F., Biediger R.J., Boatman P.D., Shindo M., Smith C.C., Kim S. (1994). First total synthesis of taxol. 2. Completion of the C and D rings. J. Am. Chem. Soc..

[50] Altshuler O., Abu-Abied M., Chaimovitsh D., Shechter A., Frucht H., Dudai N., Sadot E. (2013). Enantioselective effects of (+)-and (−)-citronellal on animal and plant microtubules. J. Nat. Prod..

[51] Edris A.E. (2007). Pharmaceutical and therapeutic potentials of essential oils and their individual volatile constituents: A review. Phytother. Res. Int. J. Devoted Pharmacol. Toxicol. Eval. Nat. Prod. Deriv..

[52] Sitarek P., Rijo P., Garcia C., Skała E., Kalemba D., Białas A.J., Szemraj J., Pytel D., Toma M., Wysokińska H. (2017). Antibacterial, anti-inflammatory, antioxidant, and antiproliferative properties of essential oils from hairy and normal roots of *Leonurus sibiricus* L. and their chemical composition. Oxidative Med. Cell. Longev..

[53] Abdoul-Latif F.M., Elmi A., Merito A., Nour M., Risler A., Ainane A., Bignon J., Ainane T. (2022). Essential oils of *Tagetes minuta* and *Lavandula coronopifolia* from Djibouti: Chemical composition, antibacterial activity and cytotoxic activity against various human cancer cell lines. Int. J. Plant Biol..

[54] Mohamed Abdoul-Latif F., Elmi A., Merito A., Nour M., Risler A., Ainane A., Bignon J., Ainane T. (2022). Chemical Analysis of Essential Oils of *Cymbopogon schoenanthus* (L.) Spreng. and Nepeta azurea R. Br. ex Benth from Djbouti, In-Vitro Cytotoxicity against Cancer Cell Lines and Antibacterial Activities. Appl. Sci..

[55] Mohamed Abdoul-Latif F., Elmi A., Merito A., Nour M., Risler A., Ainane A., Bignon J., Ainane T. (2022). Essential Oils of *Ocimum basilicum* L. and *Ocimum americanum* L. from Djibouti: Chemical Composition, Antimicrobial and Cytotoxicity Evaluations. Processes.

[56] Elmi A., Spina R., Risler A., Philippot S., Mérito A., Duval R.E., Abdoul-Latif F.M., Laurain-Mattar D. (2020). Evaluation of antioxidant and antibacterial activities, cytotoxicity of Acacia seyal Del bark extracts and isolated compounds. Molecules.

[57] Swain S.S., Paidesetty S.K., Padhy R.N., Hussain T. (2023). Nano-technology platforms to increase the antibacterial drug suitability of essential oils: A drug prospective assessment. OpenNano.

[58] Hallin J., Bowcut V., Calinisan A., Briere D.M., Hargis L., Engstrom L.D., Laguer J., Medwid J., Vanderpool D., Lifset E. (2022). Anti-tumor efficacy of a potent and selective non-covalent KRASG12D inhibitor. Nat. Med..

[59] Valizadeh A., Khaleghi A.A., Roozitalab G., Osanloo M. (2021). High anticancer efficacy of solid lipid nanoparticles containing Zataria multiflora essential oil against breast cancer and melanoma cell lines. BMC Pharmacol. Toxicol..

[60] Abdoul-Latif F.M., Elmi A., Merito A., Nour M., Risler A., Ainane A., Bignon J., Ainane T. (2022). Essential oil of Ruta chalepensis L. from Djibouti: Chemical Analysis and Modeling of In Vitro Anticancer Profiling. Separations.

[61] Topçu G., Gören A.C., Bilsel G., Bilsel M., Çakmak O., Schilling J., Kingston D.G. (2005). Cytotoxic Activity and Essential Oil Composition of Leaves and Berries of Juniperus excelsa. Pharm. Biol..

[62] Buhagiar J.A., Podesta M.T., Wilson A.P., Micallef M.J., Ali S. (1999). The induction of apoptosis in human melanoma, breast and ovarian cancer cell lines using an essential oil extract from the conifer *Tetraclinis articulata*. Anticancer. Res..

[63] Sylvestre M., Pichette A., Longtin A., Nagau F., Legault J. (2006). Essential oil analysis and anticancer activity of leaf essential oil of *Croton flavens* L. from Guadeloupe. J. Ethnopharmacol..

[64] Gharby S., Asdadi A., Ibourki M., Hamdouch A., Ainane T., Hassani L.A.I. (2020). Chemical characterization of the essential oil from aerial parts of *Lavandula rejdalii* Upson & Jury, a medicinal plant endemic to Morocco. J. Essent. Oil Bear. Plants.

[65] De Lima V.T., Vieira M.C., Kassuya C.A.L., Cardoso C.A.L., Alves J.M., Foglio M.A., De Carvalho J.E., Formagio A.S.N. (2014). Chemical composition and free radical-scavenging, anticancer and anti-inflammatory activities of the essential oil from Ocimum kilimandscharicum. Phytomedicine.

[66] Mitropoulou G., Fitsiou E., Stavropoulou E., Papavassilopoulou E., Vamvakias M., Pappa A., Oreopoulou A., Kourkoutas Y. (2015). Composition, antimicrobial, antioxidant, and antiproliferative activity of *Origanum dictamnus* (dittany) essential oil. Microb. Ecol. Health Dis..

[67] Nikolić M., Glamočlija J., Ferreira I.C., Calhelha R.C., Fernandes Â., Marković T., Marković D., Giweli A., Soković M. (2014). Chemical composition, antimicrobial, antioxidant and antitumor activity of *Thymus serpyllum* L.; *Thymus algeriensis* Boiss. and Reut and *Thymus vulgaris* L. essential oils. Ind. Crops Prod..

[68] Russo A., Formisano C., Rigano D., Senatore F., Delfine S., Cardile V., Rosselli S., Bruno M. (2013). Chemical composition and anticancer activity of essential oils of Mediterranean sage (*Salvia officinalis* L.) grown in different environmental conditions. Food Chem. Toxicol..

[69] Ainane T., Abdoul-Latif F.M., Baghouz A., El Montassir Z., Attahar W., Ainane A., Giuffrè A.M. (2023). Correction: Essential oils rich in pulegone for insecticide purpose against legume bruchus species: Case of *Ziziphora hispanica* L. and *Mentha pulegium* L. AIMS Agric. Food.

[70] Sylvestre M., Pichette A., Lavoie S., Longtin A., Legault J. (2007). Composition and cytotoxic activity of the leaf essential oil of *Comptonia peregrina* (L.) Coulter. Phytother. Res. Int. J. Devoted Pharmacol. Toxicol. Eval. Nat. Prod. Deriv..

[71] Cole R.A., Bansal A., Moriarity D.M., Haber W.A., Setzer W.N. (2007). Chemical composition and cytotoxic activity of the leaf essential oil of *Eugenia zuchowskiae* from Monteverde, Costa Rica. J. Nat. Med..

[72] Hou J., Sun T., Hu J., Chen S., Cai X., Zou G. (2007). Chemical composition, cytotoxic and antioxidant activity of the leaf essential oil of Photinia serrulata. Food Chem..

[73] Abdoul-Latif F.M., Ainane A., Merito A., Ainane T. (2022). Chemical composition and biological activities of essential oils from Djibouti. J. Anal. Sci. Appl. Biotechnol..

[74] Gudi V.A., Singh S.V. (1991). Effect of diallyl sulfide, a naturally occurring anti-carcinogen, on glutathione-dependent detoxification enzymes of female CD-1 mouse tissues. Biochem. Pharmacol..

[75] Girola N., Figueiredo C.R., Farias C.F., Azevedo R.A., Ferreira A.K., Teixeira S.F., Capello T.M., Martins E.G., Matsuo A.L., Travassos L.R. (2015). Camphene isolated from essential oil of *Piper cernuum* (Piperaceae) induces intrinsic apoptosis in melanoma cells and displays antitumor activity in vivo. Biochem. Biophys. Res. Commun..

[76] Arunasree K.M. (2010). Anti-proliferative effects of carvacrol on a human metastatic breast cancer cell line, MDA-MB 231. Phytomedicine.

[77] Suhail M.M., Wu W., Cao A., Mondalek F.G., Fung K.M., Shih P.T., Fang Y.-T., Woolley C., Young G., Lin H.-K. (2011). Boswellia sacra essential oil induces tumor cell-specific apoptosis and suppresses tumor aggressiveness in cultured human breast cancer cells. BMC Complement. Altern. Med..

[78] Qurishi Y., Hamid A., Zargar M.A., Singh S.K., Saxena A.K. (2010). Potential role of natural molecules in health and disease: Importance of boswellic acid. J. Med. Plants Res..

[79] Queiroz R.M.D., Takiya C.M., Guimarães L.P.T.P., Rocha G.D.G., Alviano D.S., Blank A.F., Alviano C.S., Gattass C.R. (2014). Apoptosis-inducing effects of *Melissa officinalis* L. essential oil in glioblastoma multiforme cells. Cancer Investig..

[80] Sheikh B.Y., Sarker M.M.R., Kamarudin M.N.A., Mohan G. (2017). Antiproliferative and apoptosis inducing effects of citral via p53 and ROS-induced mitochondrial-mediated apoptosis in human colorectal HCT116 and HT29 cell lines. Biomed. Pharmacother..

[81] Thomas M.L., De Antueno R., Coyle K.M., Sultan M., Cruickshank B.M., Giacomantonio M.A., Giacomantonio C.A., Duncan R., Marcato P. (2016). Citral reduces breast tumor growth by inhibiting the cancer stem cell marker ALDH1A3. Mol. Oncol..

[82] Naz F., Khan F.I., Mohammad T., Khan P., Manzoor S., Hasan G.M., Lobb K.A., Luqman S., Islam A., Ahmad F. (2018). Investigation of molecular mechanism of recognition between citral and MARK4: A newer therapeutic approach to attenuate cancer cell progression. Int. J. Biol. Macromol..

[83] Fayard E., Tintignac L.A., Baudry A., Hemmings B.A. (2005). Protein kinase B/Akt at a glance. J. Cell Sci..

[84] Seal S., Chatterjee P., Bhattacharya S., Pal D., Dasgupta S., Kundu R., Mukherjee S., Bhattacharya S., Bhuyan M., Bhattacharyya P.R. (2012). Vapor of volatile oils from *Litsea cubeba* seed induces apoptosis and causes cell cycle arrest in lung cancer cells. PLoS ONE.

[85] Wu C.C., Chung J.G., Tsai S.J., Yang J.H., Sheen L.Y. (2004). Differential effects of allyl sulfides from garlic essential oil on cell cycle regulation in human liver tumor cells. Food Chem. Toxicol..

[86] Hoesel B., Schmid J.A. (2013). The complexity of NF-κB signaling in inflammation and cancer. Mol. Cancer.

[87] Dehne N., Mora J., Namgaladze D., Weigert A., Brüne B. (2017). Cancer cell and macrophage cross-talk in the tumor microenvironment. Curr. Opin. Pharmacol..

[88] Ben-Neriah Y., Karin M. (2011). Inflammation meets cancer, with NF-κB as the matchmaker. Nat. Immunol..

[89] Hassan S.B., Gali-Muhtasib H., Göransson H., Larsson R. (2010). Alpha terpineol: A potential anticancer agent which acts through suppressing NF-κB signalling. Anticancer. Res..

[90] Deeb S.J. (2019). Enhancement of Cell Death by Linalyl Acetate and [alpha]-Terpineol through Targeting the Nuclear Factor-[kappa] B Activation Pathway in Human Colon Cancer Cells-by Sally Joseph Deeb. Ph.D. Thesis.

[91] Feng S.S., Chien S. (2003). Chemotherapeutic engineering: Application and further development of chemical engineering principles for chemotherapy of cancer and other diseases. Chem. Eng. Sci..

[92] Chari R.V. (2008). Targeted cancer therapy: Conferring specificity to cytotoxic drugs. Acc. Chem. Res..

[93] Çetinus E., Temiz T., Ergül M., Altun A., Çetinus Ş., Kaya T. (2013). Thyme essential oil inhibits proliferation of DLD-1 colorectal cancer cells through antioxidant effect. Cumhur. Med. J..

[94] Yang Y., Yue Y., Runwei Y., Guolin Z. (2010). Cytotoxic, apoptotic and antioxidant activity of the essential oil of Amomum tsao-ko. Bioresour. Technol..

[95] Mesa-Arango A.C., Montiel-Ramos J., Zapata B., Durán C., Betancur-Galvis L., Stashenko E. (2009). Citral and carvone chemotypes from the essential oils of Colombian Lippia alba (Mill.) NE Brown: Composition, cytotoxicity and antifungal activity. Memórias Inst. Oswaldo Cruz.

[96] Frank M.B., Yang Q., Osban J., Azzarello J.T., Saban M.R., Saban R., Ashley R.A., Welter J.C., Fung K.-M., Lin H.-K. (2009). Frankincense oil derived from Boswellia carteri induces tumor cell specific cytotoxicity. BMC Complement. Altern. Med..

[97] Silva S.L.D., Chaar J.D.S., Figueiredo P.D.M.S., Yano T. (2008). Cytotoxic evaluation of essential oil from Casearia sylvestris Sw on human cancer cells and erythrocytes. Acta Amaz..

[98] Silva S.L.D., Figueiredo P.M., Yano T. (2007). Cytotoxic evaluation of essential oil from Zanthoxylum rhoifolium Lam. leaves. Acta Amaz..

[99] Amiel E., Ofir R., Dudai N., Soloway E., Rabinsky T., Rachmilevitch S. (2012). *β*-Caryophyllene, a compound isolated from the biblical balm of gilead (*Commiphora gileadensis*), is a selective apoptosis inducer for tumor cell lines. Evid.-Based Complement. Altern. Med..

[100] Sœur J., Marrot L., Perez P., Iraqui I., Kienda G., Dardalhon M., Meunier J.-R., Averbeck D., Huang M.-E. (2011). Selective cytotoxicity of *Aniba rosaeodora* essential oil towards epidermoid cancer cells through induction of apoptosis. Mutat. Res./Genet. Toxicol. Environ. Mutagen..

[101] Ni X., Suhail M.M., Yang Q., Cao A., Fung K.M., Postier R.G., Woolley C., Young G., Zhang J., Lin H.-K. (2012). Frankincense essential oil prepared from hydrodistillation of *Boswellia sacra* gum resins induces human pancreatic cancer cell death in cultures and in a xenograft murine model. BMC Complement. Altern. Med..

[102] Rabi T., Bishayee A. (2009). d-Limonene sensitizes docetaxel-induced cytotoxicity in human prostate cancer cells: Generation of reactive oxygen species and induction of apoptosis. J. Carcinog..

[103] Legault J., Pichette A. (2007). Potentiating effect of *β*-caryophyllene on anticancer activity of *α*-humulene, isocaryophyllene and paclitaxel. J. Pharm. Pharmacol..

[104] Lustberg M.B. (2012). Management of neutropenia in cancer patients. Clin. Adv. Hematol. Oncol. HO.

[105] Zhuang S.R., Chen S.L., Tsai J.H., Huang C.C., Wu T.C., Liu W.S., Tseng H.C., Lee H.S., Huang M.C., Shane G.T. (2009). Effect of citronellol and the Chinese medical herb complex on cellular immunity of cancer patients receiving chemotherapy/radiotherapy. Phytother. Res. Int. J. Devoted Pharmacol. Toxicol. Eval. Nat. Prod. Deriv..

[106] Carnesecchi S., Bras-Gonçalves R., Bradaia A., Zeisel M., Gossé F., Poupon M.F., Raul F. (2004). Geraniol, a component of plant essential oils, modulates DNA synthesis and potentiates 5-fluorouracil efficacy on human colon tumor xenografts. Cancer Lett..

[107] Ainane A., Abdoul-Latif F.M., Abdoul-Latif T.M., Ainane T. (2020). Evaluation of biological activities of two essential oils as a safe environmental bioinsecticides: Case of *Eucalyptus globulus* and *Rosmarinus officinalis*. Przegląd Nauk. Inżynieria I Kształtowanie Środowiska.

[108] Vieira A., Heidor R., Cardozo M.T., Scolastici C., Purgatto E., Shiga T.M., Barbisan L.F., Ong T.P., Moreno F.S. (2011). Efficacy of geraniol but not of *β*-ionone or their combination for the chemoprevention of rat colon carcinogenesis. Braz. J. Med. Biol. Res..

[109] Srivastava J.K., Gupta S. (2006). Tocotrienol-rich fraction of palm oil induces cell cycle arrest and apoptosis selectively in human prostate cancer cells. Biochem. Biophys. Res. Commun..

[110] Karamysheva A.F. (2008). Mechanisms of angiogenesis. Biochemistry.

[111] Janmey P.A., Kinnunen P.K. (2006). Biophysical properties of lipids and dynamic membranes. Trends Cell Biol..

[112] Ainane A., Abdoul-Latif F.M., Mohamed J., Attahar W., Ouassil M., Shybat Z.L., El Yaacoubi A., Ainane T. (2021). Behaviour desorption study of the essential oil of *Cedrus atlantica* in a porous clay versus insecticidal activity against *Sitophilus granarius*: Explanation of the phenomenon by statistical studies. Int. J. Metrol. Qual. Eng..

[113] Maddocks-Jennings W., Wilkinson J.M. (2004). Aromatherapy practice in nursing: Literature review. J. Adv. Nurs..

[114] Okigbo R.N., Anuagasi C.L., Amadi J.E. (2009). Advances in selected medicinal and aromatic plants indigenous to Africa. J. Med. Plants Res..

[115] Talbi M., Saadali B., Boriky D., Bennani L., Elkouali M.H., Ainane T. (2016). Two natural compounds—A benzofuran and a phenylpropane–from *Artemisia dracunculus*. J. Asian Nat. Prod. Res..

[116] Spreckelmeyer S., Orvig C., Casini A. (2014). Cellular transport mechanisms of cytotoxic metallodrugs: An overview beyond cisplatin. Molecules.

[117] Sullivan L.B., Chandel N.S. (2014). Mitochondrial reactive oxygen species and cancer. Cancer Metab..

[118] Jaafari A., Mouse H.A., M’Bark L.A., Tilaoui M., Elhansali M., Lepoivre M., Aboufatima R., Melhaoui A., Chait A., Zyad A. (2009). Differential antitumor effect of essential oils and their major components of *Thymus broussonettii*: Relationship to cell cycle and apoptosis induction. Herba Pol..

[119] Gedara S.R. (2008). Terpenoid content of the leaves of Thymus algeriensis Boiss. Mansoura J. Pharm. Sci..

[120] Andonova T., Muhovski Y., Apostolova E., Naimov S., Petkova Z., Teneva O., Dimitrova-Dyulgerova I. (2023). Koelreuteria paniculata Seed Oil—A Rich Natural Source of Unsaturated Fatty Acids and Phytocompounds with DNA Protective Potential. Foods.

[121] Zeytinoğlu M., Aydin S., Öztürk Y., Başer K.H.C. (1998). Inhibitory effects of carvacrol on DMBA induced pulmonary tumorigenesis in rats. ACTA Pharm. Sci..

[122] Özkan A., Erdoğan A. (2011). A comparative evaluation of antioxidant and anticancer activity of essential oil from Origanum onites (Lamiaceae) and its two major phenolic components. Turk. J. Biol..

[123] Abusnina A., Alhosin M., Keravis T., Muller C.D., Fuhrmann G., Bronner C., Lugnier C. (2011). Down-regulation of cyclic nucleotide phosphodiesterase PDE1A is the key event of p73 and UHRF1 deregulation in thymoquinone-induced acute lymphoblastic leukemia cell apoptosis. Cell. Signal..

[124] Carnesecchi S., Langley K., Exinger F., Gosse F., Raul F. (2002). Geraniol, a component of plant essential oils, sensitizes human colonic cancer cells to 5-fluorouracil treatment. J. Pharmacol. Exp. Ther..

[125] Polo M.P., De Bravo M.G. (2006). Effect of geraniol on fatty-acid and mevalonate metabolism in the human hepatoma cell line Hep G2. Biochem. Cell Biol..

[126] Cardozo M.T., de Conti A., Ong T.P., Scolastici C., Purgatto E., Horst M.A., Bassoli B.K., Moreno F.S. (2011). Chemopreventive effects of *β*-ionone and geraniol during rat hepatocarcinogenesis promotion: Distinct actions on cell proliferation, apoptosis, HMGCoA reductase, and RhoA. J. Nutr. Biochem..

[127] Chidambara K.N.M., Jayaprakasha G.K., Shivappa M., Mantur B.S.P. (2012). Citrus monoterpenes: Potential source of phytochemicals for cancer prevention. Emerging Trends in Dietary Components for Preventing and Combating Disease.

[128] Elegbede J.A., Maltzman T.H., Elson C.E., Gould M.N. (1993). Effects of anticarcinogenic monoterpenes on phase II hepatic metabolizing enzymes. Carcinogenesis.

[129] Pattanayak M., Seth P.K., Smita S., Gupta S.K. (2009). Geraniol and limonene interaction with 3-hydroxy-3-methylglutaryl-CoA (HMG-CoA) reductase for their role as cancer chemopreventive agents. J. Proteom. Bioinform..

[130] Chen X., Shuzo O., Li Y., Han R. (1998). Effect of d-limonene, *Salvia miltiorrhiza* and turmeric derivatives on membrane association of Ras gene product and gap junction intercellular communication. Yao Xue Xue Bao=Acta Pharm. Sin..

[131] Usta J., Kreydiyyeh S., Knio K., Barnabe P., Bou-Moughlabay Y., Dagher S. (2009). Linalool decreases HepG2 viability by inhibiting mitochondrial complexes I and II, increasing reactive oxygen species and decreasing ATP and GSH levels. Chem.-Biol. Interact..

[132] Gu Y., Ting Z., Qiu X., Zhang X., Gan X., Fang Y., Xu X., Xu R. (2010). Linalool preferentially induces robust apoptosis of a variety of leukemia cells via upregulating p53 and cyclin-dependent kinase inhibitors. Toxicology.

[133] Horváthová E., Sramková M., Lábaj J., Slamenová D. (2006). Study of cytotoxic, genotoxic and DNA-protective effects of selected plant essential oils on human cells cultured in vitro. Neuro Endocrinol. Lett..

[134] Jaafari A., Tilaoui M., Mouse H.A., M’bark L.A., Aboufatima R., Chait A., Lepoivre M., Zyad A. (2012). Comparative study of the antitumor effect of natural monoterpenes: Relationship to cell cycle analysis. Rev. Bras. Farmacogn..

[135] Ferraz R.P., Bomfim D.S., Carvalho N.C., Soares M.B., da Silva T.B., Machado W.J., Prata A.P.N., Costa E.V., Moraes V.R.S., Nogueira P.C.L. (2013). Cytotoxic effect of leaf essential oil of *Lippia gracilis* Schauer (Verbenaceae). Phytomedicine.

[136] Al-Shabanah O.A., Badary O.A., Nagi M.N., Al-Gharably N.M., Al-Rikabi A.C., Al-Bekairi A.M. (1998). Thymoquinone protects against doxorubicin-induced cardiotoxicity without compromising its antitumor activity. J. Exp. Clin. Cancer Res. CR.

[137] Effenberger-Neidnicht K., Schobert R. (2011). Combinatorial effects of thymoquinone on the anti-cancer activity of doxorubicin. Cancer Chemother. Pharmacol..

[138] Dayem A.A., Choi H.Y., Kim J.H., Cho S.G. (2010). Role of oxidative stress in stem, cancer, and cancer stem cells. Cancers.

[139] Semlali A., Contant C., Al-Otaibi B., Al-Jammaz I., Chandad F. (2021). The curcumin analog (PAC) suppressed cell survival and induced apoptosis and autophagy in oral cancer cells. Sci. Rep..

[140] Contant C., Rouabhia M., Loubaki L., Chandad F., Semlali A. (2021). Anethole induces anti-oral cancer activity by triggering apoptosis, autophagy and oxidative stress and by modulation of multiple signaling pathways. Sci. Rep..

[141] Zhang H., Tikekar R.V., Ding Q., Gilbert A.R., Wimsatt S.T. (2020). Inactivation of foodborne pathogens by the synergistic combinations of food processing technologies and food-grade compounds. Compr. Rev. Food Sci. Food Saf..

[142] Yu S., Long Y., Li D., Shi A., Deng J., Ma Y., Wen J., Li X., Zhang Y., Liu S. (2022). Natural essential oils efficacious in internal organs fibrosis treatment: Mechanisms of action and application perspectives. Pharmacol. Res..

[143] Das I., Acharya A., Berry D.L., Sen S., Williams E., Permaul E., Sengupta A., Bhattacharya S., Saha T. (2012). Antioxidative effects of the spice cardamom against non-melanoma skin cancer by modulating nuclear factor erythroid-2-related factor 2 and NF-κB signalling pathways. Br. J. Nutr..

[144] Pavithra P.S., Mehta A., Verma R.S. (2019). Essential oils: From prevention to treatment of skin cancer. Drug Discov. Today.

[145] Islam S.S., Al-Sharif I., Sultan A., Al-Mazrou A., Remmal A., Aboussekhra A. (2018). Eugenol potentiates cisplatin anti-cancer activity through inhibition of ALDH-positive breast cancer stem cells and the NF-κB signaling pathway. Mol. Carcinog..

[146] Kunnumakkara A.B., Sailo B.L., Banik K., Harsha C., Prasad S., Gupta S.C., Bharti A.C., Aggarwal B.B. (2018). Chronic diseases, inflammation, and spices: How are they linked?. J. Transl. Med..

[147] Attahar W., Mohamed Abdoul-Latif F., Mohamed J., Ainane A., Ainane T. (2021). Antimicrobial and antioxidant activities of *Trigonella foenum-graecum* essential oil from the region of settat (Morocco). Pharmacologyonline.

[148] Deb D.D., Parimala G., Devi S.S., Chakraborty T. (2011). Effect of thymol on peripheral blood mononuclear cell PBMC and acute promyelotic cancer cell line HL-60. Chem.-Biol. Interact..

[149] Crowell P.L. (1997). Monoterpenes in breast cancer chemoprevention. Breast Cancer Res. Treat..

[150] Giudice A., Montella M. (2006). Activation of the Nrf2–ARE signaling pathway: A promising strategy in cancer prevention. Bioessays.

[151] Jana S., Mandlekar S. (2009). Role of phase II drug metabolizing enzymes in cancer chemoprevention. Curr. Drug Metab..

[152] Kim S.H., Park E.J., Lee C.R., Chun J.N., Cho N.H., Kim I.G., Lee S., Kim T.W., Park H.H., So I. (2012). Geraniol induces cooperative interaction of apoptosis and autophagy to elicit cell death in PC-3 prostate cancer cells. Int. J. Oncol..

[153] Ouassil M., Mohamed Abdoul-Latif F., Attahar W., Ainane A., Ainane T. (2021). Chemical composition of bay laurel and rosemary essential oils from morocco and their antifungal activity against fusarium strains. Pharmacologyonline.

[154] Kumar S., Mathew S.O., Aharwal R.P., Tulli H.S., Mohan C.D., Sethi G., Bishayee A. (2023). Withaferin A: A Pleiotropic Anticancer Agent from the Indian Medicinal Plant *Withania somnifera* (L.) Dunal. Pharmaceuticals.

[155] Ainane A., Cherroud S., El Kouali M., Abba E.H., Ainane T. (2020). Chemical compositions, insecticidal and antimicrobial activities of two moroccan essential oils of Citrus limonum and *Syzygium aromaticum*. Pharmacologyonline.

[156] Lesgards J.F., Baldovini N., Vidal N., Pietri S. (2014). Anticancer activities of essential oils constituents and synergy with conventional therapies: A review. Phytother. Res..

[157] El Omari N., Bakrim S., Bakha M., Lorenzo J.M., Rebezov M., Shariati M.A., Aboulaghras S., Balahbib A., Khayrullin M., Bouyahya A. (2021). Natural bioactive compounds targeting epigenetic pathways in cancer: A review on alkaloids, terpenoids, quinones, and isothiocyanates. Nutrients.

[158] Rigo A., Ferrarini I., Lorenzetto E., Darra E., Liparulo I., Bergamini C., Sissa C., Cavalieri E., Vinante F. (2019). BID and the *α*-bisabolol-triggered cell death program: Converging on mitochondria and lysosomes. Cell Death Dis..

[159] Martin N., Zhu K., Czarnecka-Herok J., Vernier M., Bernard D. (2023). Regulation and role of calcium in cellular senescence. Cell Calcium.

[160] Tsujimoto Y., Shimizu S. (2005). Another way to die: Autophagic programmed cell death. Cell Death Differ..

[161] Horvitz H.R. (1999). Genetic control of programmed cell death in the nematode *Caenorhabditis elegans*. Cancer Res..

[162] Fearnhead H.O., Dinsdale D., Cohen G.M. (1995). An interleukin-1*β*-converting enzyme-like protease is a common mediator of apoptosis in thymocytes. FEBS Lett..

[163] Tsuruo T., Naito M., Tomida A., Fujita N., Mashima T., Sakamoto H., Haga N. (2003). Molecular targeting therapy of cancer: Drug resistance, apoptosis and survival signal. Cancer Sci..

[164] Vara J.Á.F., Casado E., de Castro J., Cejas P., Belda-Iniesta C., González-Barón M. (2004). PI3K/Akt signalling pathway and cancer. Cancer Treat. Rev..

[165] Bouyahya A., Belmehdi O., Benjouad A., El Hassani R.A., Amzazi S., Dakka N., Bakri Y. (2020). Pharmacological properties and mechanism insights of Moroccan anticancer medicinal plants: What are the next steps?. Ind. Crops Prod..

[166] Khoo B.Y., Chua S.L., Balaram P. (2010). Apoptotic effects of chrysin in human cancer cell lines. Int. J. Mol. Sci..

[167] Ainane T., Abdoul-Latif F.M., Shybat Z.L., Mohamed J., Ainane A. (2021). Antifungal activity of essential oil of *Pistacia atlantica* against *Ascochyta rabiei* and its correlation with antioxidant activity. Pharmacologyonline.

[168] Yin Q.H., Yan F.X., Zu X.Y., Wu Y.H., Wu X.P., Liao M.C., Deng S.-W., Yin L.-L., Zhuang Y.-Z. (2012). Anti-proliferative and pro-apoptotic effect of carvacrol on human hepatocellular carcinoma cell line HepG-2. Cytotechnology.

[169] Rajput S., Kumar B.P., Dey K.K., Pal I., Parekh A., Mandal M. (2013). Molecular targeting of Akt by thymoquinone promotes G1 arrest through translation inhibition of cyclin D1 and induces apoptosis in breast cancer cells. Life Sci..

[170] Tundis R., Loizzo M.R., Bonesi M., Menichini F., Dodaro D., Passalacqua N.G., Statti G., Menichini F. (2009). In vitro cytotoxic effects of *Senecio stabianus* Lacaita (Asteraceae) on human cancer cell lines. Nat. Prod. Res..

[171] Banerjee S., Azmi A.S., Padhye S., Singh M.W., Baruah J.B., Philip P.A., Sarkar F.H., Mohammad R.M. (2010). Structure-activity studies on therapeutic potential of Thymoquinone analogs in pancreatic cancer. Pharm. Res..

[172] Sethi G., Ahn K.S., Aggarwal B.B. (2008). Targeting nuclear factor-κB activation pathway by thymoquinone: Role in suppression of antiapoptotic gene products and enhancement of apoptosis. Mol. Cancer Res..

[173] Yazan L.S., Ng W.K., Al-Naqeeb G., Ismail M. (2009). Cytotoxicity of thymoquinone (TQ) from Nigella sativa towards human cervical carcinoma cells (HeLa). J. Pharm. Res..

[174] Zhang X.J., Jia S.S. (2016). Fisetin inhibits laryngeal carcinoma through regulation of AKT/NF-κB/mTOR and ERK1/2 signaling pathways. Biomed. Pharmacother..

[175] Martelli A.M., Nyåkern M., Tabellini G., Bortul R., Tazzari P.L., Evangelisti C., Cocco L. (2006). Phosphoinositide 3-kinase/Akt signaling pathway and its therapeutical implications for human acute myeloid leukemia. Leukemia.

[176] Frauwirth K.A., Riley J.L., Harris M.H., Parry R.V., Rathmell J.C., Plas D.R., Elstrom R.L., June C.H., Thompson C.B. (2002). The CD28 signaling pathway regulates glucose metabolism. Immunity.

[177] Monirujjaman M.D., Ferdouse A. (2014). Metabolic and physiological roles of branched-chain amino acids. Adv. Mol. Biol..

[178] Patra K.C., Hay N. (2014). The pentose phosphate pathway and cancer. Trends Biochem. Sci..

[179] Abdoul-Latif F.M., Ainane A., Oumaskour K., Boujaber N., Mohamed J., Ainane T. (2021). Effect of atlas cedar essential oil against pathogenic medical bacterial strains-in vitro test. Pharmacologyonline.

[180] Abildgaard C., Guldberg P. (2015). Molecular drivers of cellular metabolic reprogramming in melanoma. Trends Mol. Med..

[181] Sun H., Wang Z., Sebastian Yakisich J. (2013). Natural products targeting autophagy via the PI3K/Akt/mTOR pathway as anticancer agents. Anti-Cancer Agents Med. Chem. (Former. Curr. Med. Chem.-Anti-Cancer Agents).

[182] Abdoul-Latif F.M., Ainane A., Oumaskour K., Boujaber N., Mohamed J., Ainane T. (2021). In vitro antidiabetic activity of essential oil of two species of *Artemisia*: *Artemisia heba-alba asso* and *Artemisia ifranensis*. Pharmacologyonline.

[183] Ramamurthy S., Ronnett G.V. (2006). Developing a head for energy sensing: AMP-activated protein kinase as a multifunctional metabolic sensor in the brain. J. Physiol..

[184] Chartoumpekis D.V., Kensler T.W. (2013). New player on an old field; the keap1/Nrf2 pathway as a target for treatment of type 2 diabetes and metabolic syndrome. Curr. Diabetes Rev..

[185] Ribas V., García-Ruiz C., Fernández-Checa J.C. (2016). Mitochondria, cholesterol and cancer cell metabolism. Clin. Transl. Med..

[186] Tomko A.M., Whynot E.G., Ellis L.D., Dupré D.J. (2020). Anti-cancer potential of cannabinoids, terpenes, and flavonoids present in cannabis. Cancers.

[187] Ballou L.R., Laulederkind S.J., Rosloniec E.F., Raghow R. (1996). Ceramide signalling and the immune response. Biochim. Biophys. Acta (BBA)-Lipids Lipid Metab..

[188] Ainane T., Abourriche A., Kabbaj M., Elkouali M., Bennamara A., Charrouf M., Lemrani M. (2014). Biological activities of extracts from seaweed *Cystoseira tamariscifolia*: Antibacterial activity, antileishmanial activity and cytotoxicity. J. Chem. Pharm. Res..

[189] Maltese W.A., Defendini R., Green R.A., Sheridan K.M., Donley D.K. (1985). Suppression of murine neuroblastoma growth in vivo by mevinolin, a competitive inhibitor of 3-hydroxy-3-methylglutaryl-coenzyme A reductase. J. Clin. Investig..

[190] Brown M.S., Goldstein J.L. (1980). Multivalent feedback regulation of HMG CoA reductase, a control mechanism coordinating isoprenoid synthesis and cell growth. J. Lipid Res..

[191] Karabín M., Hudcová T., Jelínek L., Dostálek P. (2016). Biologically active compounds from hops and prospects for their use. Compr. Rev. Food Sci. Food Saf..

[192] Sharifi-Rad J., Sureda A., Tenore G.C., Daglia M., Sharifi-Rad M., Valussi M., Tundis R., Sharifi-Rad M., Loizzo M.R., Ademiluyi A.O. (2017). Biological activities of essential oils: From plant chemoecology to traditional healing systems. Molecules.

[193] Kumar M., Tomar M., Amarowicz R., Saurabh V., Nair M.S., Maheshwari C., Sasi M., Prajapati U., Hasan M., Singh S. (2021). Guava (*Psidium guajava* L.) leaves: Nutritional composition, phytochemical profile, and health-promoting bioactivities. Foods.

[194] Fenley A.T., Anandakrishnan R., Kidane Y.H., Onufriev A.V. (2018). Modulation of nucleosomal DNA accessibility via charge-altering post-translational modifications in histone core. Epigenetics Chromatin.

[195] Bowman G.D., Poirier M.G. (2014). Post-translational modifications of histones that influence nucleosome dynamics. Chem. Rev..

[196] Mariño-Ramírez L., Kann M.G., Shoemaker B.A., Landsman D. (2005). Histone structure and nucleosome stability. Expert Rev. Proteom..

[197] Santos C.R., Schulze A. (2012). Lipid metabolism in cancer. FEBS J..

[198] Alam A., Jawaid T., Alsanad S.M., Kamal M., Balaha M.F. (2023). Composition, Antibacterial Efficacy, and Anticancer Activity of Essential Oil Extracted from *Psidium guajava* (L.) Leaves. Plants.

[199] Chinni S.R., Li Y., Upadhyay S., Koppolu P.K., Sarkar F.H. (2001). Indole-3-carbinol (I3C) induced cell growth inhibition, G1 cell cycle arrest and apoptosis in prostate cancer cells. Oncogene.

[200] Yang Y.C.S., Ko P.J., Pan Y.S., Lin H.Y., Whang-Peng J., Davis P.J., Wang K. (2021). Role of thyroid hormone-integrin αvβ3-signal and therapeutic strategies in colorectal cancers. J. Biomed. Sci..

[201] Di Martile M., Garzoli S., Ragno R., Del Bufalo D. (2020). Essential oils and their main chemical components: The past 20 years of preclinical studies in melanoma. Cancers.

[202] Santoro A., Zhao J., Wu L., Carru C., Biagi E., Franceschi C. (2020). Microbiomes other than the gut: Inflammaging and age-related diseases. Seminars in Immunopathology.

[203] Gurib-Fakim A. (2006). Medicinal plants: Traditions of yesterday and drugs of tomorrow. Mol. Asp. Med..

[204] Tajana A. (1991). Stereoselective Pharmacokinetics. New Trends in Pharmacokinetics.

[205] Labiris N.R., Dolovich M.B. (2003). Pulmonary drug delivery. Part I: Physiological factors affecting therapeutic effectiveness of aerosolized medications. Br. J. Clin. Pharmacol..

[206] Menn J.J. (1978). Comparative aspects of pesticide metabolism in plants and animals. Environ. Health Perspect..

[207] Singletary K. (2010). Oregano: Overview of the literature on health benefits. Nutr. Today.

[208] Evangelista L., Urso L., Caracciolo M., Stracuzzi F., Panareo S., Cistaro A., Catalano O. (2023). Fdg pet/ct volume-based quantitative data and survival analysis in breast cancer patients: A systematic review of the literature. Curr. Med. Imaging.

[209] Pati S., Irfan W., Jameel A., Ahmed S., Shahid R.K. (2023). Obesity and cancer: A current overview of epidemiology, pathogenesis, outcomes, and management. Cancers.

[210] Api A.M., Ritacco G., Hawkins D.R. (2013). The fate of dermally applied [14C] d-limonene in rats and humans. Int. J. Toxicol..

[211] Hegeman G.D. (1966). Synthesis of the enzymes of the mandelate pathway by *Pseudomonas putida* I. Synthesis of enzymes by the wild type. J. Bacteriol..

[212] Salgueiro L., Martins A.P., Correia H. (2010). Raw materials: The importance of quality and safety. A review. Flavour Fragr. J..

[213] Sinha A.K., Sharma U.K., Sharma N. (2008). A comprehensive review on vanilla flavor: Extraction, isolation and quantification of vanillin and others constituents. Int. J. Food Sci. Nutr..

[214] Valaes T. (1994). Severe neonatal jaundice associated with glucose-6-phosphate dehydrogenase deficiency: Pathogenesis and global epidemiology. Acta Paediatr..

[215] Fenichel G.M. (2009). Clinical Pediatric Neurology: A Signs and Symptoms Approach.

[216] Alvarez-Diez T.M., Zheng J. (2004). Mechanism-based inactivation of cytochrome P450 3A4 by 4-ipomeanol. Chem. Res. Toxicol..

